# CleavPredict: A Platform for Reasoning about Matrix Metalloproteinases Proteolytic Events

**DOI:** 10.1371/journal.pone.0127877

**Published:** 2015-05-21

**Authors:** Sonu Kumar, Boris I. Ratnikov, Marat D. Kazanov, Jeffrey W. Smith, Piotr Cieplak

**Affiliations:** 1 Sanford Burnham Medical Research Institute, La Jolla, California, United States of America; 2 Institute for Information Transmission Problems, Russian Academy of Science, Moscow, Russia; National Center for Scientific Research Demokritos, GREECE

## Abstract

CleavPredict (http://cleavpredict.sanfordburnham.org) is a Web server for substrate cleavage prediction for matrix metalloproteinases (MMPs). It is intended as a computational platform aiding the scientific community in reasoning about proteolytic events. CleavPredict offers *in silico* prediction of cleavage sites specific for 11 human MMPs. The prediction method employs the MMP specific position weight matrices (PWMs) derived from statistical analysis of high-throughput phage display experimental results. To augment the substrate cleavage prediction process, CleavPredict provides information about the structural features of potential cleavage sites that influence proteolysis. These include: secondary structure, disordered regions, transmembrane domains, and solvent accessibility. The server also provides information about subcellular location, co-localization, and co-expression of proteinase and potential substrates, along with experimentally determined positions of single nucleotide polymorphism (SNP), and posttranslational modification (PTM) sites in substrates. All this information will provide the user with perspectives in reasoning about proteolytic events. CleavPredict is freely accessible, and there is no login required.

## Introduction

Proteolysis is an important posttranslational modification that involves irreversible hydrolysis of peptide bonds by proteinases. Proteolytic processing has a regulatory role in almost all biological pathways, including cell proliferation, cell death, and blood coagulation [1]. Proteinases identify their substrates with a high degree of specificity. Accurate identification of candidate substrates for proteinases has important implication for understanding the roles of these enzymes in physiological and pathological processes as well as for designing pharmacological intervention approaches. Identification of proteolytic substrates depends on a number of factors. One important factor is the primary substrate specificity, which is defined by a specific amino acid sequence in a substrate that is recognized by the active site of a given proteinase. The efficiency of a cleavage event is also related to the structural properties of the cleavage site. The cleavage site needs to be accessible at the protein surface. Recently, it has been shown that this property as measured by an absolute solvent accessibility index is essential for a proteolytic event to occur [2]. However, cleavage sites that are hidden in native proteins can become accessible as a result of unfolding, allosteric effects, and other proteolytic activity. The efficiency of a cleavage event is also related to the secondary structure of a cleaved amino acid sequence. However, recent statistical analysis of CutDB, the proteolytic event database [3], demonstrated that proteolytic events were uniformly distributed among three types of secondary structures, although with some enrichment in loops. Cleavages in α-helices were found to be relatively abundant in regions apparently prone to unfolding, while cleavages in β-structures tended to be located at the periphery of β-sheets [2]. Other obvious prerequisites for cleavage to occur are co-localization and co-expression. A proteolytic event is not possible if both substrate and proteinase are not in the same compartment of the cell and if they enter the same cell compartment at different times.

The majority of human proteinases have multiple (hundreds of) protein targets. For more than 550 known human proteinases, the potential range of normal and pathological proteolytic events is vast. Proteinases participate in a multitude of biological functions including cell cycle progression [4], cell differentiation [5], cell migration [6], tissue remodelling [7], cholesterol metabolism [8], blood coagulation [9] and apoptosis [10]. Given such widespread importance, it is not surprising that proteinases represent a significant fraction of all druggable targets [11], and that they are driving factors in diseases like emphysema [11], thrombosis [12], arthritis [13], Alzheimer’s [14] metastatic cancer [15], as well as those mediated by viral and bacterial pathogens [16–18].

Among all proteinases, extracellular proteinases matrix metalloproteinases, play a key role in degrading extracellular proteins that help the cell to communicate with its surroundings and function normally. They are important from the physiological, pathological, and pharmaceutical points of view [7, 19, 20]. Vertebrate MMPs have distinct but often overlapping substrate specificities. They can cleave essential extracellular matrix proteins, and, as such, they are highly regulated. The 23 human MMPs can be segregated into three groups; secreted proteinases, proteinases with a transmembrane domain, and proteinases anchored to the membrane with a GPI-linkage. Thus, every MMP is poised to modify interactions between cells, and between cells and the extracellular matrix. So, it is not surprising that MMPs are involved in tumor biology, synaptic plasticity, pulmonary disease, arthritis, atherosclerosis, and sepsis, along with many others [5, 21–27]. In pathological conditions MMPs can play a destructive role, e.g. in rheumatoid and osteoarthritis [28] by degrading key constituents of the extracellular matrix [29–33].

However, even in cases where considerable effort has been devoted to the study of an MMP, our understanding of the fundamental principles that determine their substrates and biological roles remains unclear. For example, MMPs are critical for angiogenesis [22]. Experimentally induced corneal angiogenesis is lacking in MMP-14 deficient mice [34] and is significantly diminished in the MMP-2 knockouts [35]. In a mouse model of retinal regeneration after injury, neovascularization is diminished in both MMP-2 and MMP-9 deficient mice, while it is almost absent in the double MMP-2/MMP-9 knockouts [36]. Inhibition of MMPs using synthetic and endogenous inhibitors has also been shown to down regulate tumor angiogenesis, which is indispensable for tumor development [37–42]. But what are the substrates for these proteinases in angiogenesis? Is a single substrate responsible for the effects of each MMP, or do they cleave distinct substrates?

Similarly, MMPs have a role at every stage of progression of atherosclerosis [43, 44]. MMPs promote matrix invasion by macrophages [45–47] and angiogenesis into vulnerable plaques [48]. These actions contribute to plaque formation and destabilization. On the other hand, MMP-2, 9, and 14 contribute to vascular smooth muscle cell migration and proliferation, thus stabilizing the plaques by increasing its thickness [49].

Recently, it was determined that MMPs (e.g. MMP 2, 3, 9, 14, 26) could be shuttled between cellular compartments. These “moonlighting” enzymes can target not only extracellular but also wide range of intracellular proteins [50, 51].

Understanding how MMP enzymes work and what their proteolytic networks are is of great importance to biologists. However, identification of their individual substrates is a more challenging task because of overlapping specificities of MMPs. Experimental discovery of proteinase targets is time- and resource- consuming. To facilitate this process, we have implemented an *in silico* method for predicting substrates for 11 out of the total 25 human MMPs. This method is available on-line via the publicly accessible CleavPredict Web server (http://cleavpredict.sanfordburnham.org). On this server, we used the enzyme-specific PWMs as a primary way to predict positions of scissile bonds in protein substrates. The PWMs have been derived based on the cleavage preferences determined from a high-throughput phage display experiment [52, 53]. An efficient and reliable tool for substrate prediction should take into account a number of factors that, if only considered together, define proper conditions for matching substrates with proteinases. We expect that screening the prediction hits using multiple lines of independent qualifiers for proteolytic events is less likely to return a false positive. To augment the predictive ability of the PWMs to recognize positions of scissile bonds, the server provides additional information and evidences (“yes” or “no” filters) for accepting the potential substrate candidate. These include secondary structural elements [54, 55], solvent exposure, presence of signal peptide, and co-expression and co-localization of the proteinase and the potential substrate. Likelihood of the proteolytic event is low if a proteinase and a substrate enter the same subcellular compartment at different times of the cell life cycle. The data on proteinase temporal behaviour can be assessed using the data of gene co-expression. If both proteinase and its substrate are localized in the same subcellular compartment, the substrate can be classified as a potential strong candidate for further experimental verification. In addition, the server reports the presence of SNPs, with available disease annotation, and PTMs in the substrate that could interfere with the proteolysis process. The analysis of SNPs is helpful in determining whether a new cleavage site was created in the disease protein or the existing (in norm) cleavage site was removed by mutation. Similarly, the presence of PTMs may fundamentally alter the function of the protein including availability of the cleavage site to proteolysis. The CleavPredict server also offers a comparison of our predictions with information contained in CutDB database [3], provides virtual mass spectrum based on predicted cleavage pattern, and finally displays predicted cleavage positions, together with SNPs and PTMs sites on the substrate’s structure, if it is only available in the PDB [56].

In summary, the CleavPredict is a useful platform for reasoning about proteolytic events. It can be used as a discovery tool for formulating hypotheses that could be subsequently tested experimentally and conversely, it can be used for interpreting experimental findings, as has been already done in several projects [57–61]. Currently, CleavPredict is devoted to recognition of cleavage sites for MMPs, but it will be extended to other proteinases in the future. To our knowledge, none of the existing prediction methods incorporates all factors described here into the process of proteolytic substrate prediction for MMPs. The PROSPER webserver is another available prediction tool, which was developed by Pike *et al*. [62]. It offers the recognition of cleavage sites only for four MMPs (e.g. MMP-2, -9, -3, -7) and the batch mode functionality is not available.

## Materials and Methods

### Derivation of PWMs for predicting cleavage sites

The CleavPredict uses PWMs as a primary mechanism for cleavage prediction. We determined PWMs for the following enzymes: MMP-2, -3, -8, -9, -10, -14, -15, -16, -24, -17, and -25. We selected MMPs representing four main groups of enzymes according to the phylogenetic tree that was already published in our [52, 53] paper. Namely, we selected MMPs belonging to: a) a group containing simple hemopexin domain (MMP-3, MMP-8, MMP-10), b) gelatin binding MMPs (MMP-2, MMP-9), c) transmembrane MMPs (MMP-14, MMP-15, MMP-16, MMP-24), and d) GPI-linked MMPs (MMP-17, MMP-25). The PWMs were derived based on statistical analysis of enzyme specific substrates selected from phage display libraries. We used about 300 substrates for each MMP (S1 Table). Phage substrates are peptides containing constant flanking sequences (small letter) and variable six amino acid sequences (capital letters): ~ggsgPSA-LDAtasgaet~ (dash denominates the cleavage position) [53, 59].

The primary cleavage recognition method, derived for each individual MMP, has been obtained as follows. First, a set of phage substrates specific for a given MMP was selected. Then, the sequences of these substrates were aligned along the cleavage site and the frequencies (*P*(*i*_*AA*_, *j*)) of occurrence of each amino acid type, *i*_*AA*_, in each of the *j-th* position, ranging from P3 to P2’, were calculated. We use Schechter and Berger annotation for amino acid positions in substrates [63]. In Ref. [53] we demonstrated that the amino acids located at P3-P2’ positions are the most important in recognition of the substrates by MMP enzymes. Next, these frequencies at each position and amino acid were normalized by the distribution of amino acids in the set of background sequences. The background sequences comprised 766 peptides randomly selected from phage display library (S2 Table). Thus, the final PWM values for each amino acid i_AA_ at the j-th position are calculated as:

$$PWM(i_{AA},j)=\frac{P(i_{AA},j)}{P_{bckgr}(i_{AA},j)}$$ (1)

We used log_2_ values of appropriate PWM(i_AA_,j) elements, log likelihood ratios, which allows for calculating scores by adding rather than multiplying the relevant values at each position in the PWM. The primary scoring function for substrate prediction is defined in Eqs 2 and 3 as a sum of log_2_ of PWM matrix elements for *i*_*AA*_ amino acid type at the *j-th* position. Summation runs over P3-P2’ amino acid positions in the substrate:

$$Score=\sum_{j=P3}^{P2\prime }S_{j}(i_{AA})$$ (2)

where

$$S_{j}(i_{AA})=\{\begin{array}{c} \log_{2}\left(\frac{P(i_{AA},j)}{P_{bckgr}(i_{AA},j)}\right)\\ \begin{array}{ccc} \;offset, & if & P(i_{AA},j)=0 \end{array} \end{array}$$ (3)

If any element of the PWM is equal to zero, e.g. an amino acid type *i*_*AA*_ was not observed at the *j-th* position in phage substrates, then the *offset* value is used instead. From numerical point of view this is done in order to avoid calculation of infinite value of log_2_(0) and yet add the sufficient penalty to the scoring function when an amino acid type *i*_*AA*_ at *j-th* position is not observed in our learning set. The peptide bond is considered a cleavage site when the value of the score is above the *threshold* value. Both *offset* and *threshold* values are MMP specific and were derived using statistical analysis. This primary scoring function can be used to screen every peptide bond in test protein. The peptide bonds that have their score values above the *threshold* are considered to be potential cleavage sites.

The magnitudes of *offset* in Eq 3, and *threshold* are optimizable parameters of the method and are enzyme dependent. In order to establish their specific values for each MMP, we performed a series of 10-fold cross-validation calculations for a large set of *offset* and *threshold* values defined on two-dimensional grid. The optimal values of *offset* and *threshold* are those corresponding to the maximum value of F1-score. The F1 score is harmonic mean of precision and sensitivity in the machine learning theory. Our additional 10-fold cross-validation calculations demonstrated that extending the range of amino acid summation in Eq 2 beyond P3-P2’ positions does not change significantly the statistical evaluation metrics (results not shown).

We performed 10-fold cross validation calculations for each set of MMP specific substrates in two stages. In the first stage, each set was divided into two groups in approximately 2:1 ratio. The larger group of substrates was used for cross validation, e.g. involving training and validation, while the smaller set was used for independent testing of the performance of optimized scoring parameters. This smaller set is termed an “internal” test.

Next, we developed the final predictive model, in which for each MMP all available specific substrates were combined into the final training and validation sets, which were used for deriving the optimized values of *thresholds* and *offsets* parameters in 10-fold cross-validation. These are the values used for prediction cleavages in unknown targets incorporated in our web server. Combining all available data is a standard approach used to create the final prediction model for any “external” tests, as is described in Ref. [64]. The resultant log_2_ values of the MMP specific PWM matrices are provided in S3 Table. These PWMs have been applied to “external” test cases, such as evaluation of the set of substrates collected in CutDB (cutdb.burnham.org), and peptides determined as MMPs substrates by Overall *et al*. [65, 66].

## Virtual Mass Spectroscopy

We implemented an automated script for calculating mass spectrum based on a predicted set of cleavage sites. All possible mass fragments are calculated with a monoisotopic set of masses for amino acids and displayed on a separate Web page after selecting the VMS (Virtual Mass Spectroscopy) button on the result page. The intensities of the mass fragments are defined as:

Intensity(fragment)=100.0×ws1×ws2 (4)

where the *ws1* and *ws2* characterize the cleavage efficiency of the cleavage at the N- and C-terminal sides of the molecular fragment, respectively. The *ws1* and *ws2* are normalized to [0–1]range values of their respective PWM scores defined by Eq 2. For example, Eq 5 defines normalization of the *ws1* value:

$$ws1=\frac{Score-Score_{\min\_value}}{Score_{\max\_value}-Score_{\min\_value}}$$ (5)

where Score_min_value_ and Score_max_value_ are the minimum and maximum value of the score that can be obtained for a given PWM matrix. The first (N-terminal) and last (C-terminal) residues in the entire protein sequence have the *ws1* and *ws2* values assigned to 1.0.

### External programs and databases used by CleavPredict Web server

There are two main types of query inputs for testing protein substrates in the CleavPredict server: an amino acid sequence or a structure in the PDB format. In the case when an amino acid sequence is an input, the server provides information about predicted secondary structure and disorder regions for a substrate calculated by Jnet (http://www.compbio.dundee.ac.uk/www-jpred/legacy/jnet/) [67] and Disopred (http://bioinf.cs.ucl.ac.uk/psipred/) [68] programs, respectively. If a query input is a PDB structure then the secondary structure elements and solvent accessibility is calculated with the DSSP program (http://swift.cmbi.ru.nl/gv/dssp/) [69, 70]. The SignalP v.4.0 program (http://www.cbs.dtu.dk/services/SignalP/) [71] is used to predict the presence or absence of a signal peptide. The TMHMM (http://www.cbs.dtu.dk/services/TMHMM/) [72] program is applied to predict the transmembrane domains.

In the CleavPredict server, we implemented a link to COXPRESdb (http://coxpresdb.jp) [73] to determine the co-expression of a proteinase and a substrate. The average rank of this event is calculated based on the correlation score and average co-expression score. The average correlation score between the substrate and the proteinase in a gene expression pattern is retrieved and presented. We linked the Mentha (http://mentha.uniroma2.it/about.php) [74] database to our Web server. It is used to determine whether the interaction between a proteinase and a substrate is reported in literature. Each interaction is assigned a reliability score (Mentha Score) that takes into account all the supporting evidence. The information about subcellular location and positions of SNPs is retrieved from the Uniprot resource portal and from the Humsavar database (http://www.uniprot.org/docs/humsavar) [75, 76]. The experimentally known posttranslational modifications of a substrate are determined based on information available from the curated dbPTM (http://dbptm.mbc.nctu.edu.tw) [77] database.

### CleavPredict Web server implementation

The CleavPredict Web server has been implemented using Python under the web2py (http://web2py.com/book) framework and running on an Apache server on a Linux machine. The cleavage-site predictions by PWM have been automated by implementing in-house Fortran programs, integrated with Python scripts for processing. Javascript and html were used to present the final results on the user interface. The server offers two options for querying the Web server: a single substrate query, e.g., Uniprot id, Fasta sequence, PDB id, or PDB file; and a batch mode, where multiple Fasta sequences or multiple PDB files can be submitted. When a PDB structure or a Fasta sequence is submitted, the server uses standalone BLASTp against Uniprot and PDB to determine the corresponding Uniprot id. This Uniprot id is used to retrieve appropriate information about co-expression, co-localization, SNPs, PTMs, and structure of the substrate. The PDB structures are displayed at the end of user interface using GLmol (http://webglmol.sourceforge.jp). In order to display the PDB structure with all P1 positions of predicted cleavage sites and amino acids modified by the presence of SNPs and PTMs the Uniprot and PDB structure sequence numbering were mapped using the PDBSWS server (http://www.bioinf.org.uk/pdbsws/) [78].

CleavPredict is currently configured on Apache/2.2.15 (CentOS) and Python2.6.6. The scripts are written in Python and Fortran, and the server uses a web2py framework. The web2py framework allows us to separate the components of our system into the model, the view, and the controller (MVC). The model represents the data of the application, the view specifies the user interface, and the controller handles the communication among all elements of the application. Computational time required for the cleavage site prediction depends on the size of a protein substrate but usually takes less then 30 seconds for a single case input.

## Results

### Validation of the prediction method

#### Phage display substrates—internal test

We performed 10-fold cross validation calculations for each set of MMP specific substrates. Each set was divided into two groups in approximately 2:1 ratio. The larger group of substrates, which was further divided into training and validating sets, was used for 10-fold cross validation for establishing the optimal values of *offset* and *threshold* for that set. The smaller sets, not seen by 10-fold cross validation, were used for independent testing of the performance of optimized scoring parameters. The results of these internal tests for all MMPs are summarized in Table 1, while the results for 10-fold cross-validation for larger set of substrates are presented in S4 Table. The accuracy of the method exceeds 85%, true positive rate is in the vicinity of 90% and false positive rate ranges from 2.9%, for MMP-16, to 12.7% for MMP-8.

**Table 1:** Recognition of MMPs cleavage positions in the subset of phage display peptides.

Enzyme	Number of substrates		Sensitivity(TPR)(%)	Specificity (%)	Accuracy (%)	Precision(%)	MCC	FPR (%)	F1
	10F-CV set	Internal test (% of total)							
MMP-2	161	71 (31%)	81.9	96.2	93.4	84.0	0.79	3.8	0.83
MMP-9	164	98 (37%)	84.4	96.7	94.4	85.2	0.81	3.3	0.85
MMP-14	169	81 (32%)	90.7	94.9	94.0	81.5	0.82	5.1	0.86
MMP-15	159	71 (31%)	80.8	94.2	91.1	80.8	0.75	5.8	0.81
MMP-16	198	93 (32%)	88.0	97.1	95.3	88.0	0.85	2.9	0.88
MMP-24	177	97 (35%)	93.6	95.1	94.8	81.7	0.84	4.9	0.87
MMP-17	211	133 (39%)	92.4	91.4	91.6	76.3	0.79	8.6	0.84
MMP25	159	71 (31%)	84.7	92.6	90.9	76.9	0.75	7.4	0.81
MMP-3	304	87 (22%)	91.1	93.2	92.7	78.5	0.80	6.8	0.84
MMP-8	203	85 (30%)	84.7	87.3	86.9	57.1	0.62	12.7	0.68
MMP-10	170	42 (20%)	97.8	91.7	92.9	72.6	0.80	8.3	0.83

Results for the internal statistical test of the scoring function obtained for the subset of substrates not seen by 10-fold cross-validation. TPR—true positive rate, MCC—Matthews correlation coefficient, FPR—false positive rate, F1 score—harmonic mean of precision and sensitivity.

Finally all substrates specific for each MMP have been combined. Each set was divided into 10 subsets and ten training and validating cycles were performed on them to generate the final model that is used for predicting cleavages in unknown targets. The results of this training are summarized in Table 2, where last column provides the optimized values of *offset* and *threshold* for each MMP. The corresponding log_2_ of PWM matrices are presented in S3 Table. For these optimal parameters, the cross-validation achieves high accuracy (>90%) with a true-positive rate above 78% and, in almost all cases, a false-positive rate less than 10% for phage substrates (Table 2).

**Table 2:** Average values for sensitivity, specificity, accuracy, precision, Matthews correlation coefficients, false positive rate, true positive rate and optimal values for threshold and offset from the 10-fold cross-validation using the entire sets of available substrates for every MMP.

	Sensitivity (TPR)(%)	Specificity (%)	Accuracy (%)	Precision (%)	MCC	FPR(%)	F1	threshold/ offset
MMP2	90.2±8.3	94.7±2.8	93.7±1.7	81.8±8.3	0.82±0.05	5.3±2.8	0.85±0.04	0.3 / -5.0
MMP9	87.7±4.9	97.4±1.1	95.5±1.5	89.3±4.3	0.86±0.05	2.6±1.1	0.88±0.04	1.5 / -4.0
MMP14	89.8±5.3	97.1±1.0	95.5±1.2	90.0±3.2	0.87±0.03	2.9±1.0	0.90±0.03	0.5 / -5.5
MMP15	78.2±7.9	96.5±1.4	92.1±2.8	87.3±4.7	0.78±0.08	3.5±1.4	0.82±0.06	1.3 / -2.5
MMP16	93.4±3.7	95.4±1.3	95.0±1.4	83.2±3.9	0.85±0.04	4.6±1.3	0.88±0.03	0.3 / -5.0
MMP24	91.9±6.1	95.6±1.9	94.8±1.8	83.8±6.2	0.85±0.05	4.4±1.9	0.88±0.04	0.6 / -5.5
MMP17	94.2±3.0	90.5±2.4	91.4±2.0	75.7±5.5	0.79±0.05	9.5±2.4	0.84±0.04	0.6 / -5.5
MMP25	85.7±3.5	95.2±2.9	92.9±2.7	85.3±7.7	0.81±0.07	4.8±2.9	0.85±0.05	-0.3 / -6.0
MMP3	97.8±3.8	93.5±1.4	94.2±1.4	75.2±4.0	0.83±0.04	6.5±1.4	0.85±0.03	1.5 / -5.0
MMP8	80.1±10.8	90.8±2.8	89.0±3.4	63.8±8.7	0.65±0.11	9.2±2.8	0.71±0.09	1.2 / -3.0
MMP10	93.7±4.4	89.3±1.9	90.1±1.7	64.0±4.2	0.72±0.04	10.7±1.9	0.76±0.04	1.5 / -5.0

The calculations have been performed to establish the optimal values for threshold and offset parameters that are implemented in the CleavPredict web server for predicting cleavage sites in proteins. For each average value the sample standard deviations is provided. For abbreviations see Table 1.

The worst results in the above tests, e.g. characterized by the lowest F1 score, have been obtained for MMP-8 enzyme. According to our analysis the MMP-8 is characterized by the widest specificity. In S1 Fig, in Supporting Material we present the sequence logos for the substrate recognition motifs for all 11 MMPs obtained from the analysis of phage display substrates. According to the frequency of occurrence, S1 Fig, almost none of the positions in MMP-8 substrates, contributes significantly to substrate specificity contrary to what was observed for other MMPs. This makes the development of statistically robust prediction model a difficult task.

#### Analysis of proteome samples—external test

As for the first external we choose two sets of peptide substrates identified by Overall *et al*. in proteome samples for MMP-2 and MMP-9 enzymes [65, 66]. The first set of 1775 substrates for MMP-2 [66] reported in 2008, has been obtained by the proteomic identification of proteinases cleavage sites (PICS) method combined with liquid chromatography-tandem mass spectrometry (LC-MS/MS). This approach when combined with bioinformatical analysis allows for identification of the prime side sequences of the cleaved peptides. The separate bioinformatical analysis is used to establish the non-prime sequences and then to deduct the position of the cleavage sites. The duration of the enzymatic reaction was chosen to vary between 1–16 h. In 2010 Prudova and Overall [65] proposed more advanced technique called iTRAQ-TAILS, that involves isotopic labelling of substrates. This technique was applied to study specificity of several enzymes, including MMP-2 and MMP-9, for which the authors identified 201 and 19 substrates, respectively. The sensitivity of the method depends on the statistically defined reporter ion ratio cutoff for MS/MS fragmentation in samples with and without the enzyme treatment. This cutoff was uniformly established using GluC enzyme, because its specificity is well known, and cutoff ratio can be validated. Thus, both methods produced the most comprehensive to date set of well-defined peptide substrates.

**Fig 1:**
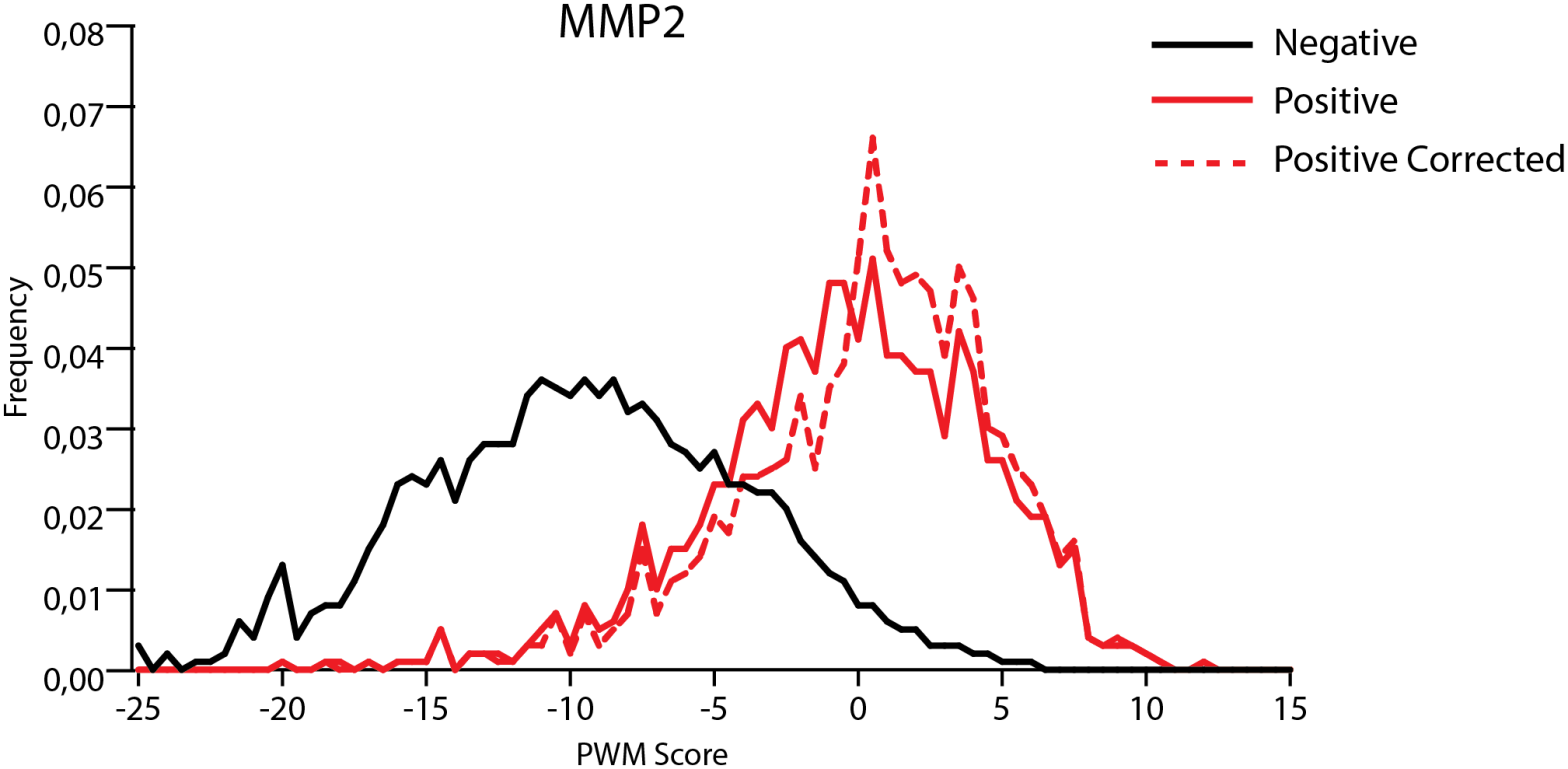
Distribution of PWM scores for peptide substrates of MMP-2 from Schilling *et al*. [66]. Red line—distribution of PWM score values for experimentally identified cleaved peptide bonds, black line—distribution of scores for all other peptide bonds. Red—dashed line represents distribution of scores for set of cleaved peptide bonds corrected by replacing poorly scored peptide bonds by those that have their scores above the threshold and were located in the vicinity of experimentally predicted positions. The separation between the cleavage site scores and the scores for other peptide bonds was subject to Kolmogorov-Smirnov test yielding *D* = 0.60 and *D* = 0.66 for red and red-dashed distributions, respectively, when tested against the black one.

**Fig 2:**
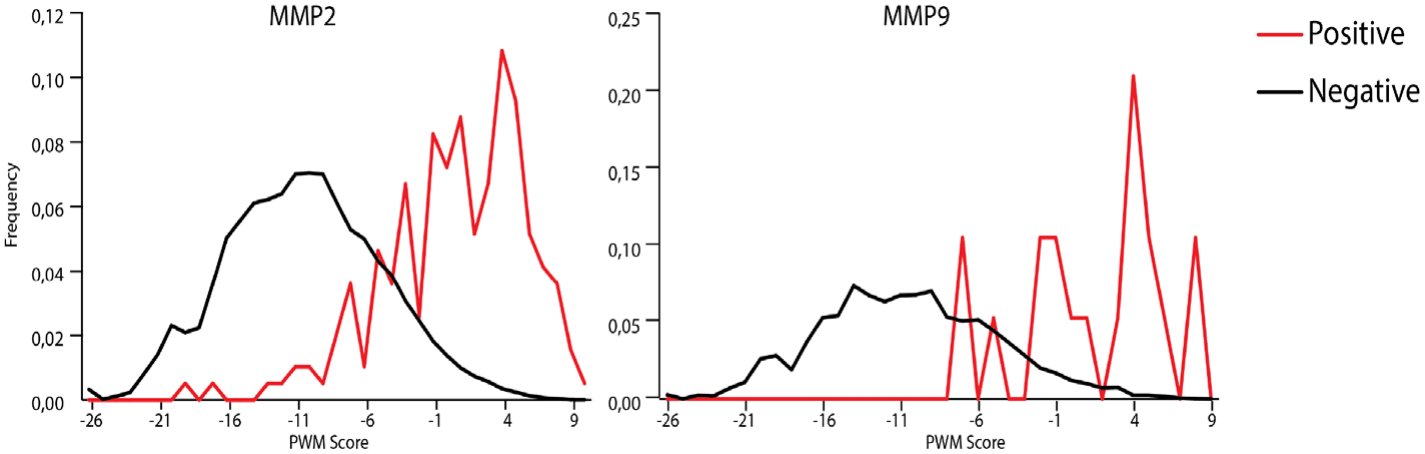
Distribution of PWM scores for peptide substrates of MMP-2 and MMP-9 from Prudova *et al*. [65]. Red and black lines are distributions of PWM score values for experimentally identified cleaved peptide bonds and for all other peptide bonds, respectively. For both MMP-2 and MMP-9 the Kolmogorov-Smirnov test yields *D* = 0.60.

We applied our prediction method to all reported peptides. The experimentally identified cleavage sites were considered to be a positive set while all other peptides bonds were negative set. We used CleavPredict to evaluate all peptide bonds in substrates and calculated the distribution of PWM score values for the positive and negative sets. The results are presented in Figs 1 and 2. In each case the separation between the scores for negative and positive sets is significant. In the two-sample Kolmogorov-Smirnov (KS) test the value of D statistic is equal to 0.6.

Experimental identification of cleavage positions in Ref. [66] depends on the accuracy of mass spectroscopy method and there is some level of ambiguity introduced by two separate bioinformatical procedures used for analysis of prime and non-prime product sequences. We hypothesize that the actual cleavage sites could be found within 1 to 4 peptides bonds next to the reported ones. Analysis of our prediction data showed it may be the case. We only include those peptide bonds that scored above the method threshold. We found 184 cleaved peptides bonds in the vicinity of experimentally identified ones that have PWM scores higher than the threshold and better match sequence patterns observed in our phage display experiment. When taking into account those new cleavage positions the discrimination between non-cleaved and cleaved peptide bonds becomes more pronounced (D value in KS test is 0.66), see Fig 1 (“corrected” curve) and Table 3. This result, of course, does not preclude the biases in our approach. Understanding them would lead to the improvement of our algorithm. Nevertheless, our results demonstrate that identification of the proper cleavage position in some substrates reported by Overall *et al*. could be revisited.

**Table 3:** Results for the external statistical test of the scoring function for the MMP-2 and MMP-9 cleavages in peptide substrates identified by Overall *et al*.

Exper. cleavage sites	Predicted cleavage sites	TPR(%)	FPR(%)	Precision(%)	Accuracy(%)	Specificity(%)	
1775	864	49.0	4.0	42.3	93.0	96.0	MMP2 Schilling, Overall, 2008 [66]
1775	1048	59.0	4.0	51.3	94.0	96.0	MMP2 Schilling, Overall, 2008 [66] (corrected)
201	120	60.0	3.0	57.1	94.0	97.0	MMP2 Prudova, Overall, 2010 [65]
19	13	68.0	2.0	72.2	96.0	98.0	MMP9 Prudova, Overall, 2010 [65]

#### CutDB—external test

**Fig 3:**
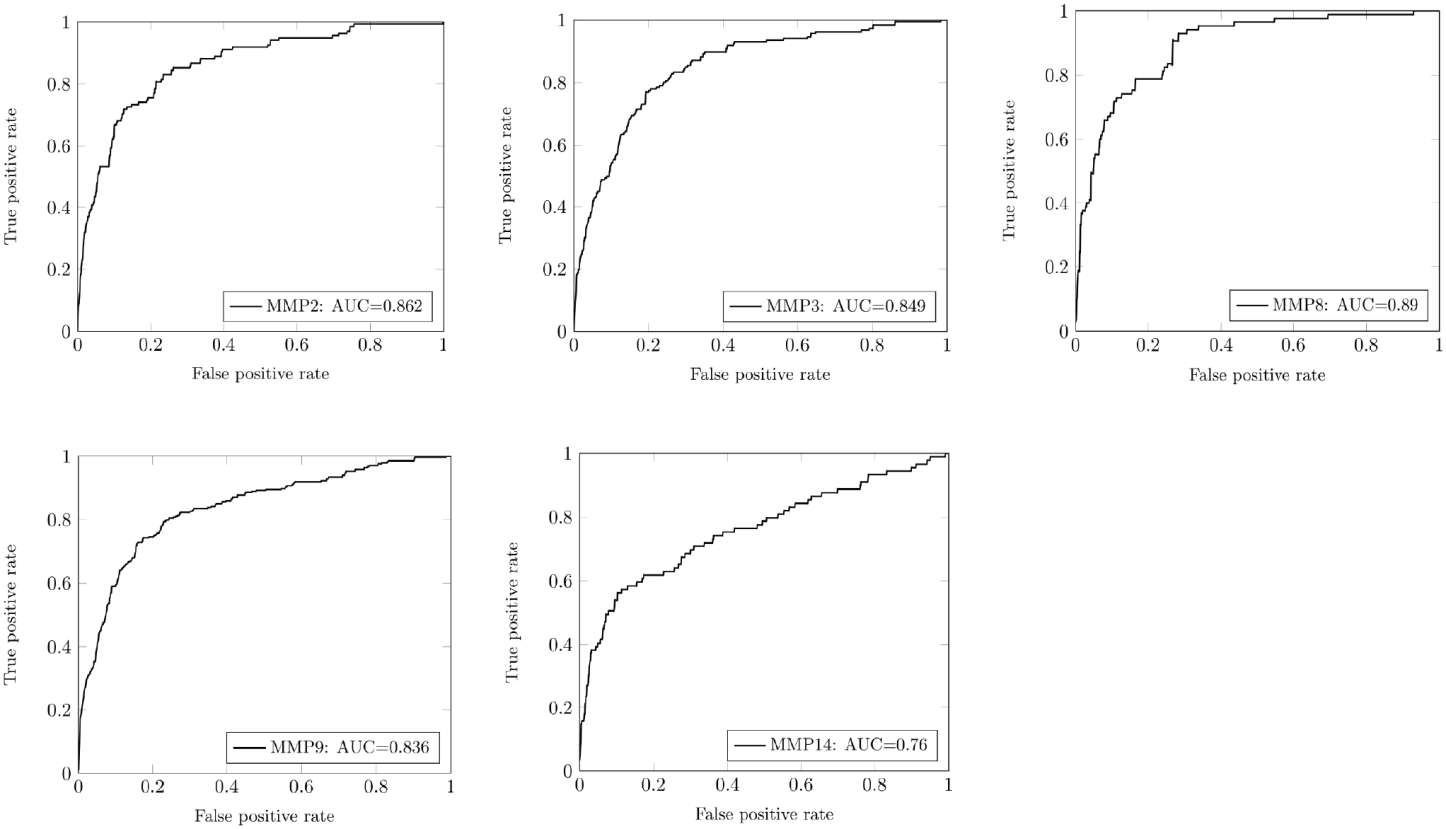
ROC curves for prediction cleavage sites in proteins collected in CutDB for MMP-2, MMP-3, MMP-8, MMP-9 and MMP-14.

Further on we validated our PWM—based scoring method in another “external” test performed on substrates collected in CutDB [3]. We selected only those MMPs for which sufficient number of cleavage events and protein substrates is available. Thus, we applied our prediction algorithm to calculate scores for cleavage sites in substrates of five MMPs including: MMP-9 (334 cleavages in 88 unique substrates), MMP-2 (135 cleavages in 50 substrates), MMP-14 (89 cleavages in 38 substrates), MMP-3 (186 cleavages in 67 proteins) and MMP-8 (85 cleavages in 26 proteins). In these calculations, the positive set constitutes the cleavage sites reported in the literature (CutDB) for appropriate protein substrates for each MMP, while for the negative set we choose peptide bonds randomly selected from the same protein substrates that are not cleaved by MMP. The ratio of positive to negative cases is 1:100. The results of our prediction calculations are collected in Table 4. It demonstrates that for experimental protein substrates the PWM approach yields the accuracy reaching the level of 70% for most MMPs, while the false-positive rate is in the range of 30%, with the exception of MMP-8 and MMP-9 for which false-positive rate is 61 and 47%, respectively. The appropriate ROC curves are presented in Fig 3. Area under the curve (AUC) (Table 4), in most cases is well above 0.8, which demonstrate a good ability of our method to discriminate between cleavable and non-cleavable peptide bonds for MMP hydrolysis. The high level of false-positive rate is not satisfactory here and substantially higher than for uniformly identified substrates by Overall *et al*., as discussed above. However, we are aware that the reported cleavage sites come from highly heterogeneous sources and may not all be entirely accurate, either because denatured proteins were used as substrates, or because when the study was performed the methods for determining the position of the cleavage sites, including mass spectroscopy, were not as robust as methods available today. What is more important, the conditions used for studying cleavage events reported in the literature could differ substantially from those used in our phage display experiment. The conditions used in our high throughput phage display experiment allow measuring important cleavage events with observed k_cat_/K_M_ values above 3000 sec^-1^M^-1^ [53]. Thus, if our predictive algorithm is sufficiently accurate, we may be able to identify the reported cleavage sites that are “suspect.”

**Table 4:** Results for the external statistical test of the scoring function for the MMPs protein substrates collected in CutDB database.

Enzyme	Cleavage sites in CutDB	Predicted cleavage sites	TPR(%)	FPR (%)	Accuracy(%)	Area Under theCurve (AUC)
MMP-2	135	115	85.2	29.4	70.7	0.862
MMP-9	344	296	88.6	46.6	53.6	0.836
MMP-14	89	61	68.5	29.0	71.0	0.760
MMP-3	186	155	83.3	28.2	71.8	0.849
MMP-8	85	83	97.6	61.0	39.0	0.890

### Description of an input and output for CleavPredict server

**Fig 4:**
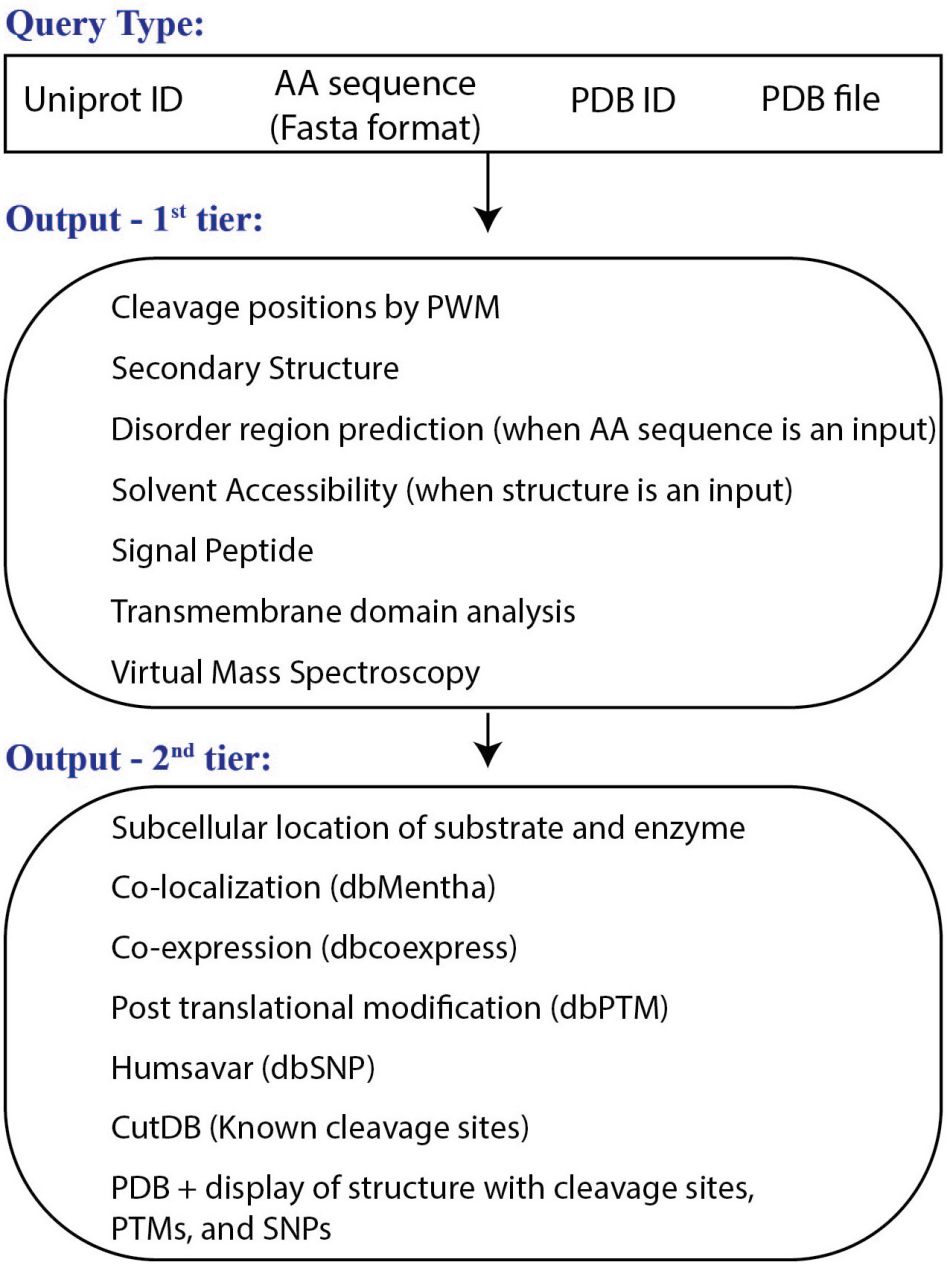
Workflow of the CleavPredict Web server. Top: the types of input queries; middle: the first tier of the output data; bottom: the second tier of the results obtained using a Uniprot id as for the input protein substrate. Blast program and mapping is used for determining the Uniprot id.

Workflow of the CleavPredict web server is presented in Fig 4.

#### User input

The user can submit either a single protein query in the interactive mode or a multi-protein query in the batch mode. In a single-protein-query mode, the input for a potential protein substrate can be provided in the form of a Uniprot accession number, a Fasta sequence, an uploaded PDB file, or a PDB id (Fig 4: Query Type). In the batch mode, the server accepts either a file containing multiple Fasta sequences, a list of multiple PDB ids (with a single space between each id), or multiple PDB files uploaded from a local computer. Once the input protein substrate(s) is/are defined, the user selects the MMP type from the list for which cleavage predictions will be calculated. The “Submit” button submits the input query for cleavage prediction calculations.

#### Outputs

**Fig 5:**
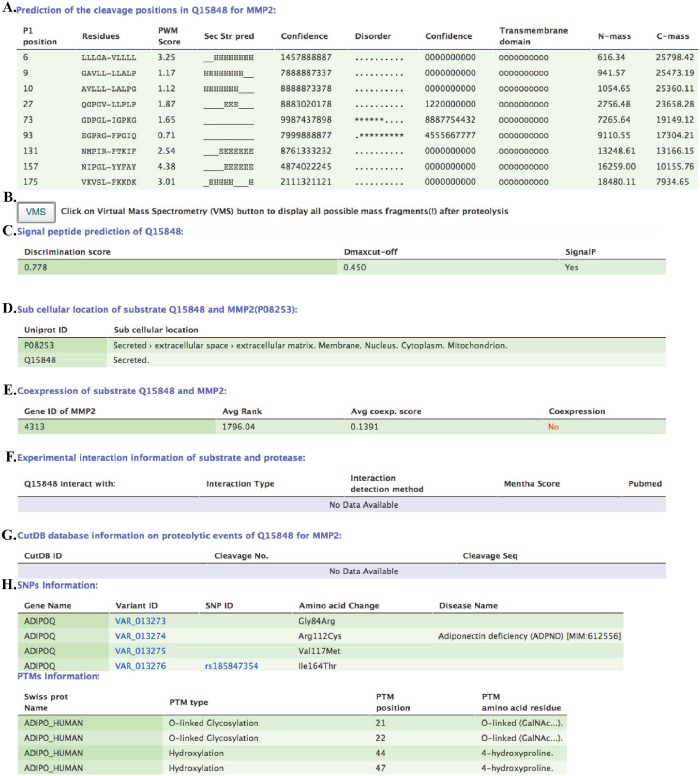
Snapshots of the result pages. As an example the prediction of the cleavage positions in Q15848 protein for MMP2 enzyme is demonstrated. This section contains information about signal peptide prediction, subcellular location, co-expression and co-localization information, known cleavages in CutDB, data on experimentally identified SNPs and PTMs, congregated into tables.

The two sections/tiers of the output data are summarized in Fig 4. All the results are shown in tabular form (see example in Fig 5). The form of the first section of the output depends on the type of input query. When a query input is in the form of a protein sequence, then the first table contains a list of predicted P1 cleavage positions, 10 amino acid sequences around the cleavage site, a PWM score, the predicted secondary structure (alphahelix; ‘H’, beta-sheet; ‘E’. loop; ‘_’) and the predicted disorder (order: ‘.’; disorder ‘*’) characterizing this region of 10 amino acids. In addition, for each of these 10 amino acid positions, the server reports confidence scores of prediction in the range of 0–9, calculated by the Jnet and the Disopred programs. The confidence score is computed for every amino acid position, as a separate number. Thus, for ten amino acids fragment the server reports ten numbers in the form of a chain of consecutive values. The table also provides information about the presence of transmembrane domain for 10 amino acids region around the cleavage site, as predicted by the TMHMM program; and N-terminal and C-terminal mass fragments resultant from each cleavage event (Fig 5, label: A).

When the PDB structure is a query input, the above-described table is partially modified. In this case, the server provides information about solvent accessibility of the cleavage site and actual secondary structure assignment, instead of predicted parameters. These properties are calculated by the DSSP program and provided in the CleavPredict result page for the region of 10 amino acids around the predicted cleavage sites.

When the Uniprot id is not provided explicitly in the input query, the BLASTp is run internally against the Swissprot database to determine the Uniprot accession number. This number is necessary to retrieve other information about the query substrate, such as co-expression, co-localization, SNPs and PTMs from appropriate databases. In the case when the PDB id is provided as an input, PDB—Uniprot id mapping is used instead of BLASTp.

The second section of the result page (Fig 5) contains information about: B) the distribution of masses after the cleavage via link to virtual mass spectroscopy results (VMS button. See Supporting Material Figure A in S1 File for example of an output from VMS); C) the presence of a signal peptide in the substrate, indicating whether it belongs to the set of secreted proteins; D) the subcellular localization of the substrate and the proteinase; E) the co-expression of the substrate and the proteinase retrieved from COXPRESSdb; F) the physical interaction between the substrate and the enzyme retrieved from the Mentha database; G) known cleavages in the query substrate retrieved from CutDB, that can be used for making comparison with CleavPredict predictions, and H) known SNPs, with disease annotation, when available, and PTMs in the substrate. Additionally, for user convenience, the server displays sequences of the query substrate in the Fasta format and color-code the predicted cleavages, SNPs, and PTMs (Supporting Material Figure B in S1 File). This information is also displayed in the PDB structure, if it is available, using the GLmol viewer.

In the batch mode, results of the calculations can be downloaded from the server or may be sent to the user’s e-mail address that is optionally provided at the time of query input.

## Conclusions and Future Developments

We will continue to integrate more proteinases into our CleavPredict Web server. This includes thrombin, furin, caspases, and others for which large set of substrates could be extracted from the literature, from the MEROPS database [79], or from our own effort aimed at high-throughput profiling of proteinases [53, 80–82]. We will work toward integrating PWMs with structural elements information into a single unified scoring function that will be used for discrimination between cleaved and non-cleaved peptide bonds. Initial work toward this goal has been already published recently [2, 54]. For many proteins, the 3D structure is not available. Instead of relying on prediction of secondary structure elements, we will incorporate a mechanism that can be used to build a homology model for the potential substrate. Homology modelling will be performed using the FFAS server (ffas.burnham.org). Our computational platform can be further extended by connecting predicted cleavage events to the chain of other events using a library of pathways and networks. The combined knowledge of the position of cleavage sites, SNPs and PTMs in the vicinity of cleavage sites, as well as knowledge of pathways and networks could be used to discover relationships between aberrant proteolytic events and potential disease or syndromes. The main problem with most, if not all, prediction methods is over-prediction of the substrates. In the case of MMPs—this problem is partially related to their broad and overlapping specificity.

We believe that CleavPredict can become a versatile hypothesis generator guiding future experiments in basic and transitional medical research. The CleavPredict has been already successfully applied to several practical scientific projects related to discovery of new MMPs substrates and helped in interpreting experimental findings [57–61].

## Supporting Information

S1 Fig Sequence logos for the substrate recognition motifs for each MMP tested in this study. Left column—frequency logos, right column—information content logos. The logos have been created using WebLogo on-line web server: weblogo.berkeley.edu [83].
(JPEG)

S1 File (A) Snapshot of the web page demonstrating the top of the scrollable table containing virtual mass spectrum data displayed after selecting the VMS button on the first result page. (B) Graphical display of the cleavage P1 positions (red), SNPs (green), and PTMs (blue).
(DOC)

S1 Table List of peptide substrates from phage display used for derivation of individual PWM matrices. (DOC)

S2 Table List of background phage display peptides. (DOC)

S3 Table Resultant log_2_ values of the PWM matrices used for substrate recognition. The header of each matrix contains the values of offset and threshold. The offset values are already incorporated into the log_2_ PWM matrices in appropriate positions where given amino acid is not observed in phage display substrates.
(DOC)

S4 Table Average values for sensitivity, specificity, accuracy, precision, Matthews correlation coefficients, false positive rate, true positive rate and optimal values for threshold and offset from the 10-fold cross-validation using approximately two-third of the entire sets (internal test) of available substrates for every MMP. The calculations have been performed to establish the optimal values for threshold and offset parameters that are implemented in the CleavPredict web server for predicting cleavage sites in proteins. For each average value the sample standard deviations is provided. For abbreviations see Table 1.
(DOC)
