# Correction: CleavPredict: A Platform for Reasoning about Matrix Metalloproteinases Proteolytic Events

**DOI:** 10.1371/journal.pone.0131952

**Published:** 2015-06-25

**Authors:** Sonu Kumar, Boris I. Ratnikov, Marat D. Kazanov, Jeffrey W. Smith, Piotr Cieplak

In the Funding statement, the name of the second funder should be “Russian Foundation for Basic Research.” The correct Funding statement is: “This work was supported by the National Institutes of Health (NIH) grant R01GM098835 (PC) and by the Russian Foundation for Basic Research grant: 15-04-06861a and Russian Academy of Sciences via program "Molecular and Cellular Biology" (MK). The funders had no role in study design, data collection and analysis, decision to publish, or preparation of the manuscript.”
