# Supplementary figures and images for: CleavPredict: A Platform for Reasoning about Matrix Metalloproteinases Proteolytic Events
Source: PLoS One. 2015 May 21;10(5):e0127877. doi: 10.1371/journal.pone.0127877 (PMC4440711; doi:10.1371/journal.pone.0127877)

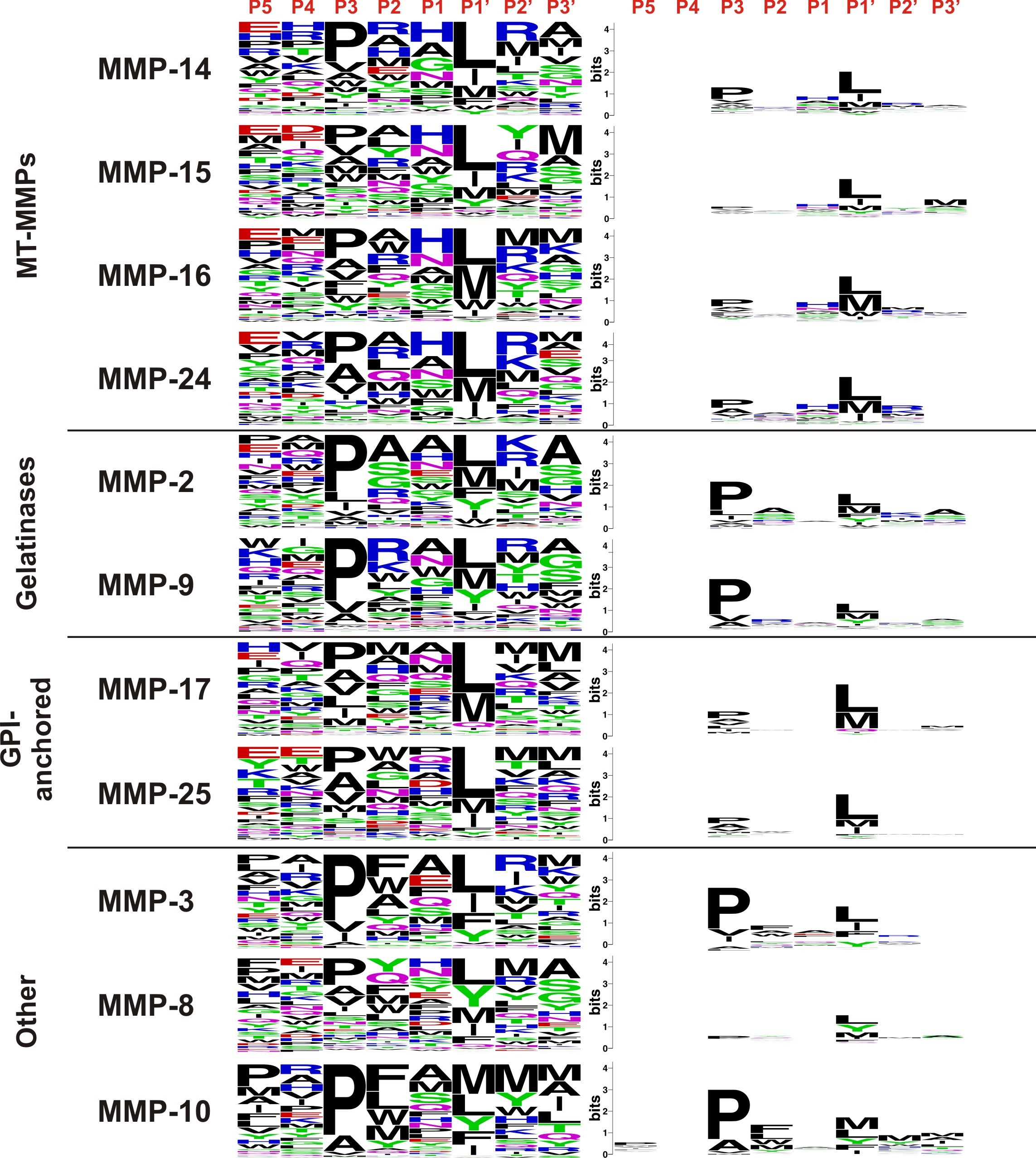

Supplement: S1 Fig — Left column—frequency logos, right column—information content logos. The logos have been created using WebLogo on-line web server: weblogo.berkeley.edu [83]. (JPEG) [file pone.0127877.s001.jpeg]

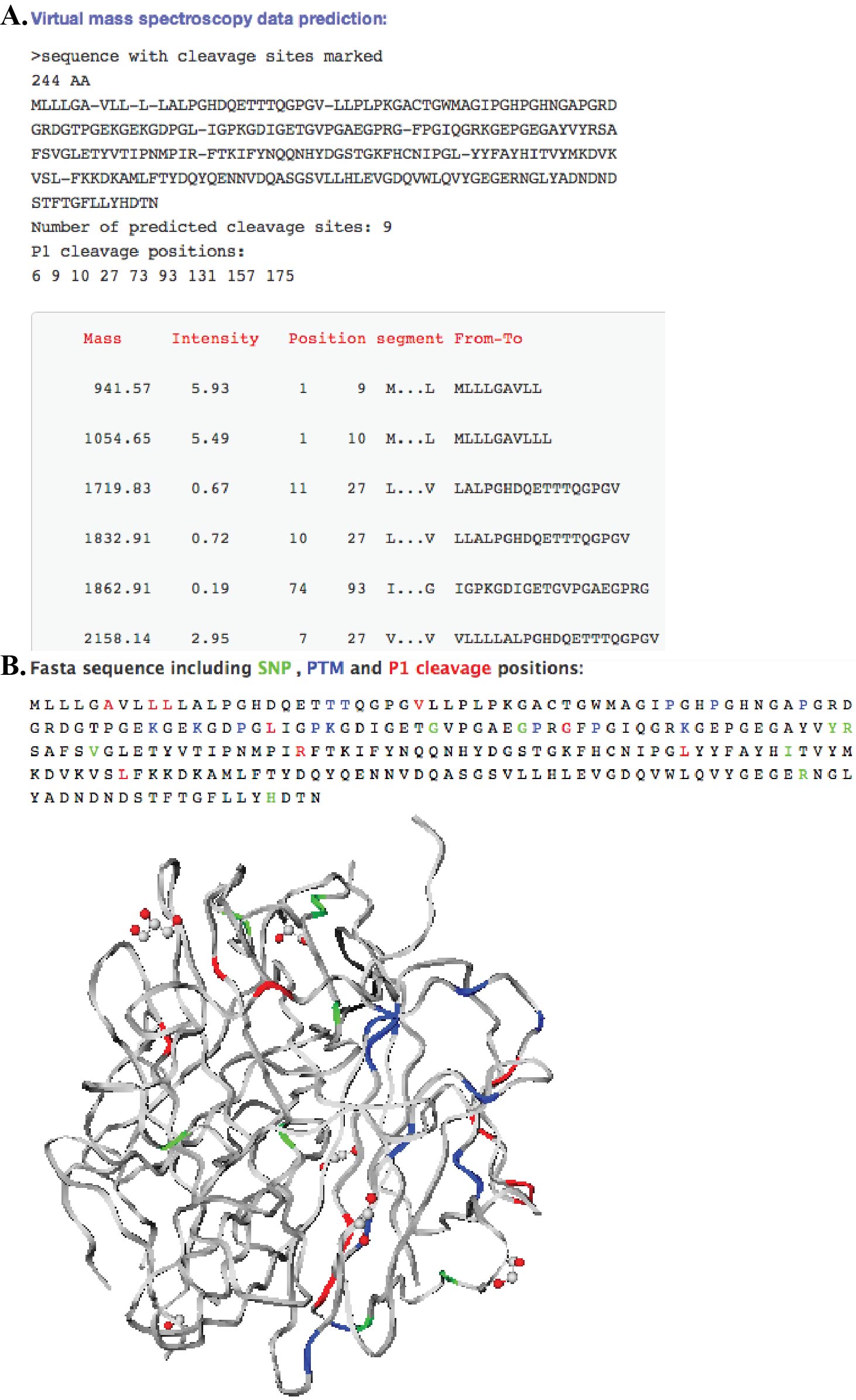

Supplement: S1 File — (A) Snapshot of the web page demonstrating the top of the scrollable table containing virtual mass spectrum data displayed after selecting the VMS button on the first result page. (B) Graphical display of the cleavage P1 positions (red), SNPs (green), and PTMs (blue). (DOC) [file pone.0127877.s002.doc]
