# Supplementary material for: CleavPredict: A Platform for Reasoning about Matrix Metalloproteinases Proteolytic Events
Source: PLoS One. 2015 May 21;10(5):e0127877. doi: 10.1371/journal.pone.0127877 (PMC4440711; doi:10.1371/journal.pone.0127877)
Supplement: S1 Table — (DOC) [file pone.0127877.s003.doc]

**S1 Table.** List of peptide substrates from phage display used for derivation of individual PWM matrices.

MMP-2

AE-MRQL AEMRQ-L A-IVRPL AIVRP-L AMPKH-L APAK-LR APAR-FT APLA-WI APLAW-I ASN-LRG ATLAA-L

ATLAA-W AT-YVAW ATYVAW- CAPTA-L DVLAR-M EAIGR-L EHPGL-L EPSA-YR ERPFF-L ESIRG-L EWPSR-M

FAPAE-V FHPSR-F FKAVA-L FPRA-FR FQIGS-L FRPVR-M FWIQS-L F-YFSNL FYFSN-L GGPST-L GHIAW-F

GIAA-MR G-IRIAD GPAP-MW GVPRA-V GVPSK-L GYFAN-L HAPSL-Y HKIAL-L H-LLSHW H-LLSKL HLLSK-L

HPRSH-L HVVAK-M HVVAQ-L H-WLGAF HW-LGAF HWLGA-F IAPAK-L -IFARSL IFARS-L IGVRA-L ILLSP-L

IQLLS-L IRIAD-L -IRLRAL IRLRA-L -ISALRK ISA-LRK ISW-MRH -IYVLSL IYVLS-L KGPSG-L KMPST-L

KQLAS-L KRFAS-L KRLAF-L K-SISNW KSISN-W KSPTG-L KYHAW-M LAPRY-W LARA-LK LDLAW-M LGPLS-L

LGPSH-Y LLQS-LR -LMHGAL LMHGA-L LMPKA-V -LMSNWR LPPLG-F LQIGH-L LQLAL-Y LSF-LKA -LYASIR

LYPQY-M -LYSTLR MALSS-L MAPFG-I MGLAN-L MPGR-LS MPLG-LR MPMGR-L MPRA-YR MRPAN-L MSLAT-L

MWVLS-L NAPNA-L N-FVGLY NFVGL-Y NGAQA-L NHLRP-L NIPTR-L N-LRSLG NPAQ-MR NPPSH-L NRPSW-V

NSPMS-L PAS-FSV PAS-LLN PAS-VRN PCPTG-L PGA-MMF PHY-LMM PIP-IAV PIPIA-V PKLAA-I PKPAR-L

PLY-YVA PMAGN-L PQLRR-F PQW-MIN PRA-VSA PRG-MKT PRN-LAV PRQ-LST PRT-LVV PRVGY-M PSA-LDA

PSG-WTA PSH-WIG PSLAE-L PSLGQ-L PTLQY-L PTPRS-Y PTWP-LS PVA-MTK PVS-MRY QMPLQ-I QRPMD-L

QRVAA-V QVGSL-M QVPRR-L RAPLP-V RAPVA-F RDLGF-L RDMSY-L RELAS-F RGPSS-Y RHLQW-L RHPSH-F

RKLAF-L R-LSALI RLSA-LI RPAG-VK RPAQ-MK RPLN-LS RPSF-LK RPTA-LL RRPVA-Y RVVAN-L R-YLSYF

RYLSY-F SHAA-LR SHAHG-L S-LFSRI S-LSSLK SLSS-LK SPLP-FI S-QLGLL SQLGL-L SRASG-Y SRLRS-L

SRMSA-L SRPRT-L SRPVY-L SSPLN-Y SSVSN-L STIAH-Y STMRY-L SVLSK-L SVPSA-I SVPST-L SYPRA-Y

TDIGE-I TMIRH-F TMLLA-L TPIE-LY TPPSS-L TPPVE-L TPRN-IK TQPSP-F TSPGL-L TVLGN-L VAW-LKH

VDVSF-L VFPQG-M VGPAF-L VIMRG-I VKPSK-L VLFGA-L VLLGN-F VLRN-LI VMFGL-L -VMGALI VMGA-LI

VMPSG-F VQLAY-F VRN-LLA VVGA-LR VWVGS-I VYPGK-M WAINA-L WDIRS-L -WIATLR WIAT-LR W-MVAFL

WNPSY-L WPLT-LR WPSE-LR WYPSK-L YEALA-F YEVGS-M Y-FKAAL YHPIS-L YKPVY-Y YQPLM-L -YTAVGY

YTAVG-Y YVLN-MK YWLNN-L

MMP-9

AFN-LYS AHPSA-L ALN-FYS A-LYAMY APRS-LV ARA-MKW ARN-LLW ASA-YRY ASAY-RY ASAYR-Y ATPES-L

AYN-FQA DPRW-LS DPWRA-L DSPLS-L EGPMR-L EPLF-LR ESIRH-L FA-LHGQ FAWS-LH FDVWM-L FEPQR-M

FIPFP-F FIPMW-S FLPRM-L FVFE-LR FVVRL-L FWASN-M GFTRW-L GGLRA-Y GKSLN-L GMVRG-V GNPYK-L

GPRS-LF GRPRS-L HAPLN-F HAPRG-S HMPLG-L HTVRG-L HTVRN-V HTVYW-M HVPRQ-V HYPLN-S HYVRA-H

IAKW-YR IAPEF-L IFPAN-H IGALA-L IGPQF-L IGVRQ-L ILVWN-Y IMHRA-L IPRP-VM IPYH-LA KAPAH-L

KDPKF-L KFPAS-L KFPWN-Y K-GFRAL KGFRA-L KHPYK-Y KIPSA-L KLGRA-L K-RVYLL KSPRP-Y KSPSL-Y

KSPVF-L KTVVW-Y -KWAYRL KW-AYRL KWAYR-L KWVYH-M LEVRR-L LIPAW-L LIPTS-L LKS-LRS LKVMN-Y

LMPVP-L -LMSHAL LPAA-LT LPAKF-Y LPALF-L -LRFMKS LRF-MKS -LRGLSG LRG-LSG -LRNLRV LRN-LRV

-LRNMEA LRN-MEA LSVFW-L -LVGRAL LVVRA-H LVWN-LW MGPWF-M MGPWG-M M-LRAMH MLRA-MH MPLL-LR

MPRS-YI -MRVLPY MRVLP-Y MSPKG-V MSPRK-F MVLG-LH MVLLH-L NGPYN-L NPLG-IR NSPSD-L NVPVW-M

PF-SNRA PFS-NRA PKF-LVM PKG-MRM PKL-LYG PKQ-LRV PKS-LVR PKW-IIQ PKW-MRH PLK-ITR PMF-LWT

PQW-ITG PRD-ILA PRF-LIG PRF-LLD PRG-QVF PRG-VTV PRL-YIG PRN-YHG PRS-HRV PRS-LKS PRVGW-L

PRW-LMS PSR-LTF PSS-LFA PTN-LRT PTWRG-L PVH-MWN PVN-LIS PWA-ITG PYS-WQV QGPWH-I QPLS-WV

QPPHA-F QPRA-IR QPRS-WR QRVRG-V QRWFA-L QSPRM-L QVAKN-F RA-MYGT RAPAS-L RATA-LY RAVLS-L

RAYG-IT REPLT-L RFPLK-V RG-LWSE RISRA-L RIWN-LM RLANA-L RLLA-MH RL-LRSQ RLLRS-Q RLPIN-L

RLPKL-L RLPYP-L RLVNW-M RMPRQ-V RMW-RAF RMWRA-F RPRG-LT RPRS-LL RPSA-MT RPSP-FW RPVH-LS

RPVN-LT RQPFL-L RQPRN-V RRPSA-L RTPKG-L RVAA-LR RVPVP-M SAIP-LW SAIPLW- SAVG-LR SDVRF-L

SGPKL-L SKPNF-L SKPWK-L SLNRA-L SLPLL-L SNPFK-Y SPRN-LR SQPIH-M SQPMA-Y SSPLA-L TAPKL-L

TGPKL-I TIPSW-S TLIRY-L TNVFW-Y TPYG-LV TSARK-L TSVSY-L TVPWS-V TVPYQ-L VAG-LYS VARP-LS

VEAVA-I VFTRN-L VILSW-Y VKG-MYS VKPSG-V VKW-LMS VLS-LQS VLT-MQG VMLRF-L VPKN-QM VPPRV-L

VPRG-VR VPRY-LR VRA-IRQ VRA-VYS VRD-LYF VRG-LFT VRG-MHG VRH-LIN VRH-VRA VRS-ITW VRW-STS

VVPNN-L VWN-LIM VWN-LMS WA-MAAL WAMAA-L WGTLA-L WLPRA-V WMPLG-M WPRE-LK WQVRY-L WRA-YTG

W-RMRAI WRMRA-I WRPFS-Y WRVHA-I WSPSS-Y W-TLLNM WTLLN-M WTPRG-N YARL-LR YGPRA-I YGPSL-L

YIPLV-Y YIPTA-F YLLKH-Y YPAG-LR YPRG-LG -YRASPL YSPMD-Y -YWASGL YWASG-L

MMP-14

AAHG-IF AAWAS-I AHG-ILS AHG-LLL AHG-LLT AK-LMAF AKLMA-F A-LRVRG AMG-LFR AN-LRSI APRF-LS

APRS-LQ APWG-IH APYNH-L ARIEY-L ARLGY-L ARPEF-L ARSYN-L ARVRH-L AVANS-L DLPAG-L DLSSN-L

D-LVGNL DLVGN-L DPYG-MR DPYS-LR DRPNY-L ELVRS-L ERFSH-L ERSLN-L ERVWA-I ESFAA-L EVARA-M

FGPYA-M FGVRN-I FHVMG-L -FLMGYL F-LMGYL FLMG-YL FLMGY-L FNPIH-L FPLM-YA -FRVRGM FVVRA-L

GAMN-LL GFPLM-L GHAYH-L GYAG-LR HH-MSNL HHMSN-L HPYS-MI HPYSM-I HQWAA-L HRHAH-L HRLGN-I

HRLLS-L HRS-LRS HRWSA-L HVHS-MR HVRH-LL HVWMN-L HYHH-LM IAKH-LM -IIYRML IIYRM-L -IRSLNT

-IRSSGL IRSSG-L -ITFRSL ITFRS-L -ITIRNL ITIRN-L -IYALTL IYA-LTL KAVNH-L KKWRG-L KMGW-LM

KTWRN-L -LAMWMR LA-MWMR LAMW-MR LDYMN-L LGPTS-L -LIINNL LIINN-L -LLSWLR LLSW-LR LN-IRGL

-LRHLSY LRH-LSY -LRIGL -LRILQ -LRVLKA -LRVWGF LSWP-LL -LTAHPL LTAHP-L -LTAIAL LTAIA-L

-LWATPH -LWRGWM LWRG-WM -MAALKR MAA-LKR -MAVRYL MAVRY-L MVQH-LI NRVEM-L NSPLS-Y NSWG-LR

NVRS-LI NY-LRAL PAA-LLG PHG-FFQ PHH-LTL PHLLH-I PKM-LTL PKMLT-L PKMSH-L PKWHG-L PLG-IRY

PLG-LSG PLLG-LW PMA-LSR PRA-LKG PRF-IRL PRM-LVL PRP-LLA PRS-LYT PRS-MLN PRVEA-L PRVHH-L

PTA-LRA PTFAH-L PVH-LLN PVYN-LR PYP-LWK PYPVP-F QAPMG-L QARG-LQ QFCSA-L QMLGF-I QPRG-LR

QT-LRSL QTLRS-L RAPLH-M R-ILRML RILRM-L RIPRF-L RKPAP-W RLGN-LW RLPVA-I RLPVH-Y RLPWS-L

RMLG-WI RMLGW-I RMLH-LR RPAD-LR RPPLA-F RQFAS-L RQWYG-I RRPTN-L RTAHN-L RTPIG-I RTPMA-M

RVAY-LS RVPYG-L RVVWG-L RVVWN-I RY-LIRL SAA-QQI SFPNP-L SHG-LRS SKMAH-L SLVRF-L SPAS-LR

SPYG-LL SQVRF-L SRA-LQM SRALQ-M STIRM-L SVHH-LI SYALH-L TFPFM-L TFWGA-L THPLG-F TLRS-LI

TRIAY-L TSPVA-L TTLRS-L TTPYH-I TTYMS-L VDIRM-L VFPMS-L VHFAS-L VIPRP-L VIRF-LR VIYGN-L

VKPWA-L VLPEP-L VLPLS-L VLRM-LS VLWRA-L VMG-IRI VNH-LMT VPPG-LR -VPRAHL VPRAH-L VRA-IIA

VRF-LMN VRG-FRT VRPRP-F VRWSW-L VVLMG-L VVRG-LS VYAH-LL VYGA-LR VYQG-LR WHPVS-L -WKTAYI

WKTAY-I -WMLSNL WM-LSNL WMLSN-L WPAG-LT WPHGS-L WPMAK-L WPNA-FL WVMES-L -YDSRGL YDSRG-L

YDVYN-L -YKVESL YKVES-L YPFGS-L YPIA-LR YPPAH-L YPRA-FS YPRN-IG YPWA-WR YPWG-FL YPWS-LS

YRA-LRL -YRLGAL YRLGA-L -YRVLHL YRVLH-L YVLN-LL YVPTG-L YWG-LKA YYPRA-L

MMP-15

AAAY-IR AEPKF-L AFRH-LR AH-LQLS AHYAS-L A-ILAHS AILAH-S AKVMS-L ALH-LKA AMP-LFY AMPLF-Y

ANW-IAM APMN-VV APVLA-L ASTLH-L AYGSH-L CVRF-LQ DALY-LR DAVYG-L DKLAS-L DNTFH-L DRQLS-L

EHAWN-L EIWLP-I ELSLA-L ERSLA-W ETMLA-L EVRF-LR FDAQS-L FDISH-L FD-LYSY FDLYS-Y FFAAY-M

FLPRR-F FMPSA-Q FPGRH-L FSAN-LK FSM-LIF FSQ-LAM FSQLA-M FWSG-LL FWSS-LR GEVFS-L GLPDF-L

GLPMH-L GMPRY-M GPSLG-I GSLAH-L HDYFN-L HG-LKSY H-LLGMS HLLG-MS HRPRS-L HRQQN-L HSPWF-L

HTSNW-L IDTRW-Y -ISLAYV ISLA-YV ISLAY-V ISVNY-L IVGM-LQ IVRY-LQ KDYLH-L KFAA-YM KFWFN-L

K-RIASF KRIAS-F KVEY-LV KWVSM-L LAWAH-I LAWG-LY -LISQWL LISQW-L -LLARMA -LLGHLY LLGH-LY

-LLLRSM -LLSYHI LLSYH-I LNPAF-L -LQGNMY LQGN-MY LQGNM-Y -LRMFEY -LRMRSF -LRNLVM LRN-LVM

-LRVKRF LS-LLAL -LSVRNV LSVRN-V -LYMILR LY-MILR -MFFYML MFFYM-L MGH-LLH MGMWH-L MKW-AAY

MKWAA-Y MLH-LKL MRP-LKL MRPLK-L MSLGN-L MSLGY-L MSPRW-L MSWSA-L MVFS-IY MVPKW-L NAIH-LQ

NHVWP-L N-LLDLN N-LTMGS N-MLGCL NN-LNLL NNLN-LL NNLNL-L NRFSN-L NS-LRQM NTSAW-L N-YIYMF

NY-IYMF NYIYM-F PAYFP-L PAY-WRT PGSYM-L PHTAS-L PKGYA-L PLG-IKS PLG-WHV PLG-YTV PSW-LYT

PSWRA-L QEPRN-I QRG-IYA -QRMASL QRMAS-L QVDY-LM QVSRH-Y RARW-LM RFPRP-I R-ILSLL RILS-LL

RILSL-L RIPAS-L RLANN-L RLRN-LV RLYD-MI RLYDM-I RN-LRLQ RP-LMSK RQLAA-I RQWRA-L RTASH-L

RTGLY-L RTMLH-M RVYD-LK RW-LRSG SEPFG-L SEWQG-L SGSRA-L SIG-LWA S-IIGWF SIIGW-F S-ILSAL

SILSA-L S-IRMFL SIRMF-L SLA-LQS S-LELYL SLE-LYL SLELY-L S-LVMPY SMGSH-L SMNS-LR SPMH-LI

SPPLA-L SPVG-FK SRAYS-L SSVWH-I STRN-LI STVYS-M -SVGLYL SVG-LYL SVGLY-L SVMRL-L SVNY-LV

TALP-LR TATNW-L TILRN-I TKMAW-L TKYAY-I TLG-WQL TLGW-QL TLGWQ-L TLMG-LY TLN-LYN TPMQ-LY

TQVFG-L TVPQM-I TVSYA-L TVTQN-L TVVAY-I VFVSN-L VHFH-LI VKAYN-L VPWGN-L VQQQG-L -VRMFNY

VRMFN-Y VRPIM-L VRY-LYG VSF-LQQ VSSYW-L VVLN-LE VWRG-ML VYS-LIK VYW-LRS WA-LRSP WAYN-MR

WDALG-I WKVSS-L WQYSN-L WVSY-LK -YCSLPL YCSLP-L YDIHF-L YDISA-L YDTQH-L YH-LRHT YMS-LIA

YS-LRMF

MMP-16

AA-LTMP AFLSH-W AG-LRMF AGPQP-L AGVRY-L AHVFA-L AMA-LKL AMPLH-M ANYSN-I AQFKH-L AQM-LRG

ARS-LKG ARS-MRL ASH-MTL ATIAH-L AVLSN-L AVP-LYR AVPWM-Y AWHN-MR CPLS-LY D-MRHLS DMRH-LS

DSLAW-L DYPLS-I EFAA-LQ EFRS-MR ELMLS-M EMSWG-L EMYGN-M ENFLH-L EPNY-LK EQAWY-L EQLSF-L

ERIYA-L EVARA-W EVRG-LR FAH-LTS FFLH-LT FLAH-IT FLH-WRS FPQS-LM FPVLS-L FQFEH-L FRN-LRI

FSARS-L FSLRQ-M GGPSM-L GKLYY-L GLGLN-L GLLLN-M GMAQY-L GMPWS-V GNVAN-L GPFL-LK GPIP-LW

GRLAS-L GSVMN-L GSVWA-L G-WWMNL GWWMN-L GYAN-LT HAHAS-L HARS-LK HG-LWSL HGLWS-L H-IWQNL

HIWQN-L HLAYH-M HLPIF-L H-LRNLE HLRN-LE HNANY-L HQN-LMA HRPWM-L HRSQG-L HSYH-LM HVSGN-L

HWPVG-L IAFSQ-L IFWH-LR IPAFN-L IPLLN-L ISRFW-L ISWLN-L IVKH-LQ KAWA-MT KGPEM-L KLAS-LR

KLKH-LQ KMFH-LI KMPSF-L KMVYM-L KSPLG-L -LISRSI -LLFSQL LLFSQ-L LLPQA-L -LLYASL LLYAS-L

-LLYMGL LLYMG-L -LMLRHL LPLN-WR LPTA-LR LPYA-LY LPYFS-M -LRHLSN LRH-LSN -LRNMSK LRN-MSK

-LRSFML LRSFM-L LSHAW-L LTPKW-I LTPRY-W LVGNS-L -LWYGHL -LWYLSA LWY-LSA -LYRHMV LYRH-MV

-MALSML MALSM-L MARF-LS MAWG-LR MELFN-L MNWES-L -MRIFSY MRIFS-Y -MRSWWL MRSWW-L -MTMRRL

NFARY-M NFRH-FR NKVYA-W NMWAH-L NRLSH-W NRPAS-W NTPNW-L NVRF-FM NVRN-LI NVSRA-M PAW-MKG

PFF-LRK PFH-LSR PFY-LRN PIA-LHW PIALH-W PKFSH-M PLG-ITL PLG-YFA PLVSW-L PMVRW-L PNY-IRA

PNY-LSM PNYLS-M PQS-WRT PQVWY-L PRA-FSV PRAFS-V PRLTH-L PRP-LYH PRVWL-L PRW-MKG PSG-MRV

PTLGY-L PVA-LYK PVFQS-L PYIKF-L QH-LRKS QH-LRVH QHWGG-L QIVRP-L QLWQ-MR QRPAQ-L QSWYG-M

QVSWA-W RAAW-LQ RAPSM-L RASH-LM RDLSH-L RFALN-L RGAN-LK RGPYH-L RGPYM-L RH-LIKV RKVRN-W

RLLLN-M RLYMN-I RMFP-ML RMLAQ-L RN-LMSV RNPEW-M RPPSS-L RPYS-IT RQLAH-I RQVRY-L RRN-LMN

RRVLM-M RSHAS-L RS-LMLR RSPLM-Y RTPVG-L RVSA-IR RYAQP-L SFGH-LM SFRG-LR SIAFA-L SIHLA-L

SIVRM-L SLFLH-M SLPRP-L SLPTS-L SLVLN-M SQPTG-L SRYLP-M STVFH-M SVWH-VR TALG-IL TFARS-L

TIPFY-M TKWLA-L TLMS-MY TMGMN-M TNPIM-L TPSA-LT TPYP-LR TRLAH-V TSFRM-L TSLAN-L TTHYS-L

TTLRW-W TVLNS-L TVPRA-W TVRS-LI TVVGW-L VEVHS-L VFW-MRG VIRWI- VKVFS-M VLG-LIG VLPRS-W

VLWAG-L VLYH-LQ VMVWN-I VMYGN-L VNARM-L VPPEA-I VRF-LKG VRLNY-L VR-RAFL VRRAF-L VRVLH-L

VVRG-LK VVYRG-L VWG-IYV VWN-MAM VYLGA-L VYRH-MR VYWGN-M WA-LKKV -WFLQKL WF-LQKL WGG-MRL

-WMASIQ WMAS-IQ -WRVFML WRVFM-L WRYSN-L WSG-MMA WSN-LMR YAN-MRR YAS-MVA YEPRN-V YFSAH-L

YKFGH-L YKPAG-L YLVRY-L YN-FRML YNFRM-L YN-WRHL YNWRH-L YPLH-LQ YRS-LTL YRS-LYS YSATN-L

YVAAP-M YWAH-MV YYG-LRA -YYLESM YYLES-M

MMP-24

AANW-FR AFH-ILG AF-LLAM AFLLA-M AFS-LMR AHA-LHL AHALH-L AHVYS-M A-IYRSM ALA-LLD ALG-LLE

ALH-LRE A-LLAFI ALLAF-I AMAHS-L ANAMN-L APAF-LK APLS-LS ARAQH-I ARN-LMA ATLRM-L AW-LKKI

AWN-MRV AYH-LQQ AYS-LRV CCSLA-L CLLAS-L DAPYF-L DILRH-L DRIRH-L DVAFH-L DVRH-IF DYARA-M

EHNLP-L ELPNF-L ELYAS-L EQLAN-M ERIWA-L ERLLH-L ESAQM-I EVSQH-L EVTMW-L FHIAH-L FHRS-IR

FIARA-L F-MYSHL FMYSH-L FSPRF-Y GHQW-LR GKPLP-W GKTWA-L G-LLQHM GLLQH-M GMILS-L GMVLN-L

GVRW-MM HAHH-LY HCL-MAL HGPMS-L HHPAA-L HKVLG-M H-LVAWL HLVAW-L HMH-MRS HWPRF-L IALS-LL

IEYLN-Y -IFINFL IFINF-L IGFAG-I IGYAW-M -ILSLPL ILSLP-L -IQLAWL IQLAW-L -IYGLRN IYG-LRN

KMPRW-I KRAAS-L -KRAYSY KSPRM-L KVLAS-L LA-LKMG LAVG-LR LAYS-LQ LGWSS-L -LKIQAL LKIQA-L

-LLGLRL LLG-LRL LNAFG-L LPALN-M LPQA-LL LPTRW-L -LRFLAM LRFLA-M -LRGLMR LRG-LMR -LRMWIR

-LVLGWL LVLG-WL LVLGW-L LVVAA-L LWPWS-F MAYQS-L MD-LLSL -MFHLNI MFHLN-I -MMSPLR MMSP-LR

MVPSA-L -MVVRHL MVVRH-L -MYLNLK MYLN-LK NAFLS-L NDLAH-I NFGRP-L NF-LRKM NFPMS-L NKTAW-L

N-LALKQ NLA-LKQ -NRPSAL NRPSA-L NTFLH-L PAA-LQS PAG-MHS PDVFA-L PGIAN-L PGVRF-L PIP-IRK

PLA-VRT PLG-IFY PLS-ILN PLS-LRS PMA-LKS PMARY-L PMG-MRN PMH-LRS PMS-LLD PMS-LRM PRA-LMV

PSPRA-I PSS-LLG PVAAY-L PVLRR-L PVPSN-M PVS-LRM PWG-LLE PYAEN-L PYN-LRG QAAFH-L QAWA-LG

QG-LKVR QG-LRMG QKYSN-L QPFA-LS QPRN-LS QRANS-I QSIA-LR QVPVF-L QVRA-LR RALA-MQ RDVLW-L

RFARY-L RFGS-LK RGPVF-L RHALH-Y RHQS-LR RKALN-L RNLAS-L RN-LRHV RPHP-LS RPMN-LR RRPAS-M

RRTAW-L RSMW-LA RTPLM-L RTYSH-L RVAH-LR RVIRH-L RVLH-LR RVLH-MF RVPRG-L RVWH-LQ RYPVN-M

RYPWA-L SAVRA-L SDIRW-L SIPNA-L SKIRF-L SKVLF-L S-LKASI SLPAH-I S-LYRAL SMPRM-L SPLS-WR

SPVW-LR SRILH-I SRPLN-L SRPRM-L SRVLP-L SRVRN-L SSPLY-L SSYAH-L STVRN-L SVARN-L SVVRM-L

SWG-LAL SYVHW-L TAVRS-L TGAWY-L TKVAW-I TLPHS-L TLTHH-L TMAHP-L TPGS-LR TRPQH-L TTALW-L

TVLRS-L TVMAF-L TVRH-LS VAS-LMG VDHAH-L VDILH-L VFALN-L VFS-LKL VFYQS-L VGHN-LR VHAVW-L

VHVHA-L VIPLM-F VIVAA-L VKAQH-L VLFH-LS VLMRG-I VLS-LRY VMHQN-L VMSLG-L VNVRN-L VPPMP-L

VPQW-IH VQLRS-L VRF-MRQ VRKGS-L VRM-LRR VTPLY-L VVAVS-L VWG-IRY VWGRA-L WAS-MRL WLAQA-L

WLN-LRS -WLYAHY WRAIS-L WVANM-I WVRN-LL YAH-MRV YAQF-LK YATWS-M -YFARQL YFARQ-L YGLH-LR

YKFFS-M YLPLL-Y YLPRA-I YMH-IKV YNVNY-L YPIMN-L YQTMA-L YRF-MTL -YRVAFL YRVAF-L YSPRF-I

YTMLH-L

MMP-17

AGLDL-L AK-LIVR ALDW-MS A-LIAND A-LQRLP A-LQVPT A-LRVQL A-LSKLR ALSK-LR A-LTLKL ALT-LKL

A-LTMPL AMN-LTL ANS-LVA APQL-IS APSS-LR APVYA-L AQA-LTL ARQ-LVL AS-LLLS ASL-LLS AS-LQML

ATASF-L AVA-LRL AVLRS-L AVTYA-L A-YIHRL AYIHR-L EKVML-M E-LRLSN EPIYN-L ERPGW-L FAA-LRA

FAAYS-L FAIS-MV F-LEFMK FLEF-MK F-LIGPM FLIGP-M F-LMSWM F-LVRSL F-LYNLS FLYN-LS FMMGN-M

FN-LQYL FNLQY-L FPFR-QL FQ-LRVF FSE-LTL FS-LRTW FTPLN-M GA-LMKL GALMK-L GE-LSFL GELSF-L

GISMY-L GLGE-LR GLGM-ML G-LGVPH G-MIAFL GMIAF-L G-MVLQL GMVLQ-L GSA-LQN GSIWK-L GTIFR-L

GT-LSKL GVFK-MR GVMS-ML GVMSM-L GVSF-LL GWILRSL HE-LSYL HELSY-L HK-LSAL HKLSA-L HLAS-QR

HLPTA-L H-MIFPL HMIFP-L HP-LEWL HPLEW-L HPTL-LK HPWL-QR HRPFG-L HVLQN-L HYPSS-L IAHQ-LK

IAMN-IL IAQ-QQV IAQQ-QV IEAWS-L IES-MYV IGL-YRT IHAWK-L ILKN-LL IMMAY-L INM-LTL IPFF-MT

IQT-MKV IR-LSML IRLSM-L ISFA-LT ISF-MSV ISPAT-L ISPML-L ISQS-LI ISY-MRI IVNAR-L KAALP-L

KASN-LY KPAS-LD KVAF-ML KVAFM-L KVHQ-ML KVNN-LV KVSE-LI LAK-LTV -LAMMMG LA-MMMG LAM-MMG

LAMM-MG -LAMNML LAMN-ML LAP-LKS LAPLK-S LAS-LRL LE-LRVN -LFRLQS LFR-LQS LGC-LKA LGH-LSV

LGM-LAT -LGRVQR LGR-VQR -LGVFKM LITGM-L -LITMGL LITMG-L -LLLTLL L-LLTLL LLLT-LL LLN-MYI

-LLSRMR L-LSRMR LLSR-MR -LMINFS -LNIERL LNIER-L LP-LMLR LPLM-LR -LQLKMM LQ-LKMM -LQMALV

LQMA-LV LQN-LML LQN-LYF LQPLT-I LQSMN-L -LTAVAL LTAVA-L -LTLLHL LTLLH-L -LVAKLI LVAK-LI

-LVLAIQ LVLA-IQ -LVLNRL LVLNR-L LWT-LMA LYG-LTL MKPVN-L M-LKLQP M-LLMKQ MLS-LVI M-MLYKL

MM-LYKL MR-LVLR MSS-LKF -MVKNLR MVKN-LR MVPHM-L -MYLQLR MY-LQLR MYLQ-LR NA-LMLV NALM-LV

NAMR-MV N-IVSML NIVSM-L N-LIYWN N-LLGKL NLLGK-L NPLP-QM N-QVWRL NQVWR-L NRIGN-F NR-LMRL

NYA-LTM NYALT-M PAP-LVL PAR-LIS PFR-LNW PGPES-L PHL-LIR PHS-MKL PHW-LNA PIS-LLG PISL-LG

P-IVMGY PIVMG-Y PKQ-LTR PLA-STV P-LMLPF PLS-QST PLT-LNS PMQR-LM PMR-LVT PRVML-L PSD-LHA

PSN-MLR PVM-LRG PVMR-MS PVVWW-Q PVY-LNL PVYLN-L PYL-ISL PYLIS-L PYT-LVM QHLAA-L QHMR-LI

QKVLN-M Q-LKVVH Q-LLLRN QPVP-ML QQMS-LR QRMDL-L QSPVQ-L QVFR-LI RA-LRVL RAQN-LL RASA-LN

RDLWK-L RDVLA-L RGMQN-L RHLAK-L RILGY-L RIYE-LW R-LLQFL R-LMNMF RLMN-MF R-LNMRL R-LSWAL

RLSWA-L R-LVGYL RLVGY-L R-LVLRV R-LVVRL RPAQD-L RPLQ-YR RPVYR-M R-QVWRL RQVWR-L R-RLQML

RR-LQML RSPLM-M RVEL-LY RVHR-LS RVLSR-L RVQR-MT RVTWA-L SAHL-LL SALA-LT SFN-LTM SFPLQ-Y

SHSN-LM S-ILYRL SILYR-L S-LRLFE S-LVLRL S-LVNLL S-MAMLA SMA-MLA SMAM-LA S-MLRLR SM-LRLR

SMLR-LR SMR-LVL SPVQ-LT SQPHL-L SRIAL-L SWS-LIL SYMFS-L TALGY-L TAPIN-L TGVSW-L TIAH-LH

TIAY-LN TIDL-LT TKVLW-L TLAA-MS TLLD-LI TMMS-LM TPLG-QI TPVGW-M TSVLA-L TTPSW-L TVWA-LT

VAGA-LY VAKP-LW VAP-LIL VFN-LAF VHY-LHR VKVVS-L VLG-LYR VLGL-YR VLHS-LR VLK-LLR VLKL-LR

VLMR-LN VLQK-LV VMMQ-LV VNL-LLM VNS-LRR VPHS-LY VPLN-VK VPSW-LS VRAHS-L VRA-LIS VRL-LKA

VRTMA-L VSISF-L VSLAY-L VTVIA-L VVHS-FC WHFGA-L WMHE-LK WSVQL-L YAPGS-M YFGN-LR YIQS-LF

Y-LYSLL YLYS-LL YPSER-L YRTHS-L YS-LIAL YYGE-LL

MMP-25

AAGW-MQ AAWG-LN AFQ-ISR AGF-MYM AGR-LRA ALD-LKR A-LHISF ALH-ISF A-LHRPG A-LREKV A-LTSRF

AN-LLYL ANL-LYL ANY-IRA APDQ-LS ARVGV-L AVSGF-L A-YTMVA AYTM-VA CAQE-LT DLPHC-L DT-LLGL

DTLLG-L EAVGN-L EAWH-LF EIAS-LS E-LAWHL ELAWH-L ESPSF-L EVWR-LH E-YKLFA EYK-LFA FAKH-LL

FA-LVAK FGASA-M F-IGLWS FIGL-WS F-LFNAV F-LVAKA FMH-IWL FRVEP-L -FTLKSL FT-LKSL FTLKS-L

FTVHM-L GQPNF-L GVVSR-L HA-LLQS HAL-LQS HGS-MVL HISWD-L HR-LMMG IGY-ISV -IHLNRL IH-LNRL

IHLNR-L -ITTGYL ITTGY-L KAPLK-L KEPAS-F KFAT-LV KIEY-LI KP-LRKL KPNR-LV KPQN-LR KPVSA-L

KSLW-LK KSPAY-L K-VLLGL KVLLG-L KWPER-L LA-LKAY -LALNLH LA-LNLH LALN-LH -LARLSM LAR-LSM

-LFINDL LFIND-L -LFMRRD L-FMRRD LF-MRRD LGW-MMA LGWR-LY -LIFSLK LIFS-LK -LMRSYI L-MRSYI

-LNSWSL LNSWS-L -LQLARR LQ-LARR -LTLALN -LYLRKN LY-LRKN MA-LTHI MGPVR-I M-LILSS ML-ILSS

MLP-FTN M-LQYVC MLQ-YVC MSF-LRL NAD-LIM NADL-IM NA-LQLY NALQ-LY N-LTFLA N-LVRVR NSHMA-L

NS-LKRR NSPQL-L PAGH-LS PAQD-LY PLH-QTK P-LRARL PLR-ARL P-LVLSY PQA-VVK PRS-LSQ PSPRY-L

PSR-ILM PTLWR-I Q-LFNVG Q-LLATK Q-LRREY Q-MVVKL QM-VVKL QNQ-LRL QPAH-LS QRVLP-L RASR-LV

RAWG-LI RAWM-MV RGVWK-L RHMLG-L R-LFWQL RLFWQ-L R-LKWNL R-LLRIV R-LMYER R-LRLAI R-LSQAL

R-LTLKW R-LVMTY RN-LGRI RNLGR-I RNVNF-L RPRG-LV RPSW-LM RRPLR-I RRVLA-L RVPKL-L RWPAW-L

SAR-LTL SD-LKSK S-FSSPL SFSSP-L SGG-LGF SGG-LGQ S-LLRSR SL-LRSR S-LNQRL S-LSISQ SLS-ISQ

S-LSVWK S-LTFNT S-MLLPL SMLLP-L SPAHG-L SPALD-M SPLD-LK SQAS-LM SQASL-M S-VMRSL SVMRS-L

SYP-LTR TGSWP-L TLAQ-IS T-LMNFL TLMN-FL TLMNF-L TMPYL-L TPSK-LY TRMAL-L TTSAS-L TTSLR-L

TVAY-YR TVSLP-L TWAKM-L TWP-LSG TWR-LML VAISR-L VGA-LNK VGM-LYN VHR-LSY VINQ-LR VKF-LFN

VKVWG-L VMK-LQL -VMMKLV VMMK-LV VMR-LVV VMY-LQV VNR-ISL VNS-LFT VPFM-IL VQL-LGS VSAR-LV

VSVDQ-L VWP-LVK VYR-LEL VYSAY-L WD-LKRS WGDR-LT WNSLN-L WR-LTVG WVGH-LS YEAHA-L YFPLD-M

YKAFT-M Y-LFRLV Y-LQFLE YLQF-LE YSALP-M YSPSL-I -YWMLPI Y-WMLPI YWMLP-I YYASH-L

MMP-3

AAVAA-L AFE-LRF AFPLS-L AFS-LRM AGPIA-L AILLA-L AIPIS-F AKPLM-L ALA-LGL ALALG-L A-LIGML

ALIGM-L A-LLFSL ALLFS-L AMPQA-I APAF-IR APAL-LV APAY-LV APFA-FK APIA-FY APIAF-Y APMT-LS

APVF-LM APVFL-M AQA-LTY AQLAS-L ARPFP-Y ARVWM-L AVAFS-L AVPLS-F AVYF-YM AYPAS-L DGIAF-Y

DLVFP-F DRPWF-L DTPFF-L EEPAS-L EFVFF-L EVAWS-I E-YIAAL EYIAA-L FAIFS-L FAPAM-L FAPAS-L

FAPLA-V FFPLQ-I FGPMS-F FGPMT-F FIAA-LS FI-IHNL FIIHN-L FKPMS-V FKVYS-L FLPFN-L FLPLS-L

FPYA-FR FVAQ-LI FYIAS-M FYVGS-L GMPQL-L GPAA-LL GPLS-FR GPMS-IR GQVFY-Y GRPHQ-F GRVFF-Y

GVFH-LI GVPLM-I GVPWR-L GVWL-LR HGPHA-L HGPMT-L HIAE-FR HIAFQ-L HLLFR-L HMPWA-I HRPWQ-F

HSIAQ-L HSPFH-L HSPFL-L HTVWH-L HVAF-LS HYVSA-L IAF-IVR IAF-VRL IAHF-MI IAL-LVS IAP-LRA

IAPLR-A IFN-LAL IGPQS-L IPFG-LT IPHS-LT IPMQ-LA IPSM-LL IPYA-FL IRIFS-L IRLFA-Y IRPLS-Y

IRPQQ-L IRPVA-L IRVWM-L ITPQS-L IYPLQ-Y KIAE-LR KPAE-LN KPFA-IK KPFD-FR KPFY-II KQVFE-L

KSPYS-L LAA-LAI LAALA-I LAPLQ-L LGH-LLG LGPFF-L LGPYF-Y LIPQS-I LIPWL-I LIPWT-I LKPLL-L

LKPQS-F LKPWL-I LLAYA-L LLIWD-L LLPFL-L LLVFN-S LLVGS-L LLVQE-L LMIHL-Y LMPFR-F LNIFA-I

LPPWT-I LQ-IAML LRPQT-L LRPYS-V LTLWA-L LTVYN-L LVFQ-LR LVPFP-F LVSE-LR LWVSE-L LYLGQ-L

LYPLA-F LYVYH-F MAIFN-L MAPAS-L MGPSA-Y MKVFQ-L MPLP-LT MPVFS-L MP-YSLI MPYS-LI MVAF-LR

MVPYA-I MWVGM-L NAPHF-I NCVFQ-L NGPLM-L NIWF-LR NIWL-LT NKPLA-V NLPWM-L NPPFG-L NRVWA-L

NSIAY-L NSPFA-H NVAFE-L NVPFR-L NWLFH-I NWVQA-L PAA-LMV PAA-LRI PAF-ILT PAP-ILS PAQ-LRL

PAS-LIR PAS-YYQ PAVYS-L PCPYS-L PFA-ILN PFA-YVA PFE-YRM PFL-LQG PFM-LLQ PFM-LVK PFP-LKW

PFQ-LST PFT-YIG PHA-LTR PHA-YFY PHAYF-Y PHQ-LRH PHVFA-L PIA-FMI PIAF-MI PIAFM-I PIA-LKL

PKAWP-L PKPMT-L PLA-LNW PLE-LRS PLF-IMR PLG-LFK PLG-LIR PLH-LLK PLHL-LK PLM-LWR PLP-LRF

PLP-LSW PLPSF-L PLQ-YLL PLS-FVI PLS-LSY PLS-LYK PLT-LKL PLT-LVV PLY-YTT PM-AYIR PMA-YIR

PMAY-IR PMPAT-I PMS-LAW PMS-LFS PMS-LHE PMT-LLS PQA-VLA PQS-LST PQY-YIL PRIWR-L PRPAA-Y

PRPEY-L PRPLQ-L PRPSS-V PSVAY-I PTPLS-F PVIYL-L PVL-LIM PVLL-IM PVPFL-I PVVFQ-L PWA-IIY

PWA-ILT PWA-VRG PWL-LVL PWVGF-L PYS-LWK PYS-YVM QHPWA-L QHVFF-L QIAF-FT Q-LIANL QLIAN-L

QPAA-LR QPLS-YV QPVFF-Y QTPLS-Y QVFL-YV QVPFA-H RAPFA-Q RAPLA-I RAPLS-L RAPLY-L RAPYA-Q

RAVAM-L REPHF-L RIPAS-L RIPWE-Y RLIGQ-I RLWQ-LR RMPYS-L RPFN-LL RPFV-LR RPLA-IR RPLS-LW

RPM-AIR RPPLS-L RPPSY-L RPWM-LI RPYA-FS RPYE-IY RQVYA-I RSPMA-F RVAFA-L RVAF-IR RVFA-LK

RVPYF-F RWVLA-L RYAFF-L SEVWQ-L SFPAA-V SGPLF-F SIPFN-I SIPMM-L SIPWE-L SIVLA-L SKPHF-L

SMPFG-L SPFL-LK SPFS-LI SPLS-IR SPQS-LS SRIAF-I SRPLA-Q SRPWS-V SRVFL-L SSPAP-L SSPFY-L

SSPLE-I STIWA-L STPLL-I STVFN-L SVISF-L SVPHS-F SVPIS-L SVPST-L S-YVLAL SYVLA-L TAPYF-L

TGPLE-L TILWA-I TIPLQ-L TIVYL-L TKPYA-F TLAWA-I TLAYA-L TLVAT-L TLVFS-L TPLS-LV TQVFA-L

TRALA-I TRIFL-L TRVFE-I TSPVA-L TTVWA-F TVFL-IR TVPLS-F TVPWN-L VFLAH-F VFQ-LVQ VGPVA-L

VGVFY-L VHPWN-L VIAFS-I VKIWA-F VKVWN-F VLPWA-L VMPAF-L VNPWS-L VPFM-LK VPFS-FR VPFY-FR

VPSF-LR VRLWM-L VRPLH-I VRPSS-F VRVWE-Y VTIWN-L VTPMM-I VVVAY-L VVYE-LK VWAFN-L VWQ-YRL

VYLAA-F VYS-LIR WAIAA-F WHPAA-L WIAT-LK WIPLG-F WKPAQ-L WLIAA-Q WLWE-LT WMPFN-F WNIGM-L

WRISF-L YAPYT-I YGLFL-Y YGPWA-L YKVLA-L YMPHQ-L YMVWS-I YNLFA-L YPWE-IR YRPQF-F YRPYF-F

YRVAA-L YVPQQ-L YVVQA-I YVWA-YR YWLAP-L YYPQS-L

MMP-8

AEGWL-M AFH-YTV AFS-LVV AIA-YLA AKNLA-M ALAFL-M AMGLG-L AMRYS-L ANA-LYG ANAYP-W ANVMF-V

APFM-LN AR-LTAL ARVFH-M ATSYL-F AVQF-LQ AVVQR-L DEPRW-L DGTLR-L DKLYE-I DSDMR-L DWIYR-L

EQVSP-L ESTLN-M FAANS-M FDPGY-I FENAN-L FF-LSSL FIAHG-L FLSMG-Y FPFH-MQ FPQH-FK FPVSS-Y

FQSLQ-I FRPMK-F FRTQF-L FSPRS-L FSVYT-M FTVFA-L FTVHP-I FVANH-F FVGMY-L FVLKN-L FVPAV-L

FVPLD-L -FWSDRL GAFY-YR GGFQL-L GGPQR-S GGYQL-Y GIMWR-I GLTLS-F GLVRY-L GMPYK-M GNKMN-Y

GTPVR-Y GVILY-V GVVLN-V GWHLS-F GWLGL-F GWNSN-L GWSHA-L GYMFT-I HAPLY-S HELFR-I HFPFF-H

HHARA-L HKLSR-L HMAWG-I HMSWS-L HPSD-LR HQF-LVG HRGYL-Y HRPWS-S HSMFF-L HWPLL-W HYGLK-Y

H-YRAAL IGTMF-L IHTSF-Y ILS-ILA ILTYS-Q IPASH-L IRPHL-L ISPTH-F ISYN-YV KLSQK-F KMSYS-L

KPLD-IR KPSS-LS K-RMWAY KSVWL-V KTKYN-L KWHFE-Y KWSLP-L LA-LVAP LAPTS-I LESYN-M LEVYE-L

LFQLN-I LGPVR-L LGSFS-Q LGVLT-I LH-IRAL LHPAL-F LKPAH-A LKTQD-L LLKHL-L L-LLAPM LLPYD-Q

LLY-LVS LMF-LLD LM-LRAR LNINY-L LNRSN-Y LN-YQFL LN-YRGP LPLS-YM LPTLK-L LR-LMWI -LRSTFY

LSF-FMA LTRYT-L LTTQR-L LTVSR-F -LWSLGL LY-QWAQ MASQS-L MDPYF-L MMPRN-Q MNAQV-Y MPVMF-L

MRTYL-L MRVGR-L MS-VRAL MTLWL-L MVPHL-W MVPVS-Y MVVDK-L M-YKGSL NGWN-LV NHIWN-L N-LRGLI

N-LRSLL N-LYGII NMQ-LRA N-MVSKL NPLS-LN NVPKF-I PAPTL-L PAPVD-L PFG-MLA PGL-LLH PGWAY-L

PHVAL-Q PILSR-M PIN-LTF PKA-LMG PLG-LWA PLG-LYV PMQ-IKS PQF-YMI PRIHT-L PRSLG-I PSF-YLT

PSGLP-Y PSINH-L PSTQR-L PSVGM-L PSWQQ-I QAVEN-F QHVWK-L QNPLS-Y QP-YLAA QQNYY-I QR-LLNL

QRPFS-A QSEYR-Y RAIYH-L RAYFM-Y RDPVE-Y REFN-LT REPYR-A RGFLE-L RIVLE-L RPEL-LV RREFL-L

RTGQH-L RTVFH-Y RTWLP-F RVASR-Y RVWD-LM RVYM-LR RWAHS-L SAN-LMG SDLQR-L SEVFM-M SFPQP-M

SGPFA-S SGSSS-L SHSWP-I SIHLS-L SKIWF-I SKIWS-L SKPYA-S SLARS-L SLE-LVS SME-LRV SMHFP-I

SN-ILGL SQA-LRH SQARW-L SQVYQ-Y SRPGE-L SRSYT-L SRTQH-Y STHMA-I SWN-LTM TANY-LR TDSYH-L

TENYA-Y TFPGK-F TKQYG-Y TLPIQ-M TNVRF-L TNYAR-L TPLR-LK TPRA-LV TPRY-LI TPVRL-L TQVAH-L

TQW-YYS TRAFR-M TRFYF-V TRVDY-L VAQY-LI VDSYS-I VEMYK-L VFQMP-L VGFSF-I VIPRF-F VKALK-M

VLASL-L VLGWL-L VMLSR-Y VNY-YRA VPGN-LF VRDFR-M VRNYG-L VRPVL-L VSRMD-L VSWD-LT VTMLT-L

VTTMH-L VTVNN-I VVGLA-M VWH-FTG VWLSS-M VYKWD-L VYPHA-L WASQT-Y WDAQS-V WE-YLGM WGPLT-M

WIPIA-L WMNAR-Y WMNQF-L WP-LRNL WR-YTAS WS-LLKY WSMN-YS WSQMP-F WT-LVAL YDANM-L YEPHL-M

YG-LHSY YGVFA-Q -YHGYSI -YLSWFM YPMK-LG YQFSH-L YQRYS-I -YRGFQL YS-LKSI YT-LYAV YVVYT-I

YYPFT-Y

MMP-10

AFVLA-Y AHPLS-L AHPMS-L AIAFN-M AIALM-M AIPLR-Y AIPSA-Y AKAFS-L ALAFA-L APLG-LY APLL-FV

ARAML-L ARLM-SL ARPLD-Y ARPLS-L ARPMS-Y ASAWL-F AVPFT-L AYPLG-Y EYALA-L FAPLT-F FAPWG-I

FEVWM-L FHPLD-L FKPLF-L FPYGQ-F FRA-FMM FRAFM-M FRPFN-L FSILQ-M FSPFS-I FTPML-M FTPMS-L

FVMWN-L FYPWS-F GLVFW-L GRPFL-L GSPFN-F GVVLH-L GYPLA-L HGPLA-F HIPWG-L HKPF-QF HKPFQ-F

HRVLL-L HVAFL-I HVPLY-F HWPLQ-M IAGLA-M IESWS-L IFPYL-Y IHAFK-L IHPMM-L IIPLQ-L IKPMN-Y

IPAFA-I IPAL-ML IPALM-L IPVFS-L IRAWL-L IRPMF-I IRPQY-Y ITPFP-Y IVPAA-F KVPMF-M LAPFR-L

LAPLG-L LAPML-M LAPYM-I LEPLA-F LGPLP-M LHPLS-L LIPLS-L LKPLS-F LLAFN-I LLA-MHL LLAMH-L

LLPFS-L LPPMS-L LRALM-L LRPFN-Y LRPLQ-M LRPSA-L LRPYM-L -LWAMSF LWAMS-F LYPLG-I LYPLS-F

LYPWS-M MAPLK-Y MGPFY-M MHPLL-I MLAFS-L MLAYH-F MMMFE-L MMVYA-L MPMMF-Y MPPLA-L MPYM-LI

MRPFL-L MRPLL-M MRPWH-L MRPYS-L MRVFH-F MVPFA-Y MVPLS-F NAPSP-F NCPFC-L NEPFY-I NGPFM-M

PAA-LLL PAALL-L PAPIF-Y PAPLT-M PAS-MSL PAW-SYR PAWS-YR PEPYL-L PFA-MFI PFPAS-L PFP-LMI

PFPLM-I PFP-MKM PFS-LTA PIA-MHL PIAMH-L PKALF-Y PKAQF-M PKPSM-M PLA-LKV PLALK-V PLA-LRA

PLPLE-Y PLS-YLT PMK-MWY PMM-LYR PMP-IML PMPIM-L PMPWQ-Y PMY-YSS PRAFS-Y PRALS-L PRAWL-I

PRGFA-L PRPIG-L PRPYS-L PSA-WSL PSAWS-L PSPFR-L PSPHS-Y PSPLQ-Y PTALE-L PTAWY-F PTPML-M

PTPWT-L PTPYP-L PVAYS-L PVPLY-I PVVFA-L PVVFY-L PWA-LYT PWP-FRI PWPFR-I PYA-WYQ QAPLS-M

QGPFN-L RGPFA-L RKPLG-L RMPLA-M RPFL-LM RPLQ-LQ RTVLY-M SHPFS-Y SNPWS-F TRSLS-M VFAFS-F

VFPLF-F VGPLA-M VIVLN-L VKPWF-Y VLPWF-M VPFN-MM VPLQ-MW VPQA-LM VPVWA-M VRAFM-M VRAYE-L

VRAYN-L VRPFA-F VRPWS-L VRPYM-Y VSPLA-M VVAWS-F VVPFP-Y WAPFL-L WGPLL-I WIPMA-I WKPLM-L

WPAFQ-L WQPLS-M YAPLH-I YFPMS-L YHPMA-Y YLVLN-L YMAQS-L YQAFA-I YSPLT-L YTALF-F YTPLA-L

YVVFS-L YWPLS-I YYAWN-L
