# Supplementary material for: CleavPredict: A Platform for Reasoning about Matrix Metalloproteinases Proteolytic Events
Source: PLoS One. 2015 May 21;10(5):e0127877. doi: 10.1371/journal.pone.0127877 (PMC4440711; doi:10.1371/journal.pone.0127877)
Supplement: S2 Table — (DOC) [file pone.0127877.s004.doc]

**S2 Table.** Background sequences used for derivation PWM matrices.

NPNLKR KAVVVA PFMVGL RKCMIS VDWSQP GSNGYQ VRSNFV VLNVPA YRHIGL ESSWLR FLRGTW MAKVNG LTMRTN TGLTQI

ERLNFK IIDNAW ALRELF DHYGKL RYLMHK WSFDRK SYLVMS KSTHWL YHAWQR RMNSDK NYLTRR PDDCTG VVCAFC RFYQGW

QTACIT SRYNLG YDFVIS RVIPGE VVWIKV ARMRGL NYSPGF DSSMGM VTSWHN INEDEN SGYVRP DWTVRI EEINAN YVDVML

WPSKWD WREKWS RSYTMI WMPQLY NTFAKG FPSKWN PYRGYG SYPKNF YPVMLF LSRPTL SLSFFT RGGTRD YATRLG GVGELW

VRYNLD TQSLWL GYRRWI WTDSPS KDAGVL LTRTTR DKVSSL EPNTSP FWDDNP HNLLVN AALLRF HTAFDN SDPRSY IDFGAV

ITLNVK LPRVLL DANYRL LYESTY TDTPSI RQKINL TTSPIR VTMRSG REAYRG ETGRVY RNKSNM AWKQQD LSHRTS ILGKVI

MGRSMG LQTALQ FPSVFS GYMTFD STTWKR VYLVSL IWSLLS AGFLLL VGKGWK FFNMAT DRRYIN HKTIWL LTNSSR SCSRQS

RITHNN TFYLAS LCTWHS DRTLWQ AMLARA RHIPLG KDHHET KPDWFR GKWIFA LKWQLA RTRVRG RIDVYL SRVYQY TGAKVL

TLSAYS VSYNSI NVAISM REVAPG TVSRVC WRQKNY IGLSQT VWENGW TGHQNR MMKVSL ILAPSA YKARTL WKKGAV GPTYVL

KTNNWV SVSMCT AMSLGL YTHQ*G FSGLTR WNYGSQ KLSAIR LRLSLA QKIFVT HDVDIW GRLFGR HSSLKR SYVAQF ALKNLF

GIFGYY DSMKSI LGNLEN NRKRIK KYGWQG LTWKYL MDLRPY LGFSRI FSVHPA HLTARI MDSFSR DSSCHA YSSTSD NFRSLD

HIKSGV HMYAKW KWYMLQ VSGYGT ALNPTP VTGGQT FTAARF PGMRVM FYWYQD QTWRSS ASRFMK ERTDWL RNEWTK PEMKFN

HMLSVL NMIGRS VRVSSE NRFARG LQRLHS FVSMAG YVLRWL RLAMER DSTLRP RDRPFF FSLHFK RYVTMF RMPNYW RISLSA

VRPLFV PLFIVW HIARVS VELRSM KSPRAR SSFPMA RITHNN FGRVAA WTDRTR LHKKPG KWQVHE LNQSSR PLAFRV KGRYAY

VRWPFE FGNKKA STFVMG KVNRFI YLAGSR RTILTF IPLRIV VKHKNL SPPNWS TVRLGR TGPINY SLGYVL SHVQTR RLWLQG

NTSVLC RFGKHV HQMNNG QYSWLL IWDRKL DGQDYK ELWHRQ VFMNSL TGIIEP YREGYL GLMELG NSRWAL AALHQK DVYRWR

SIGKLV QSGLDK HSRYMV PTFTRH SLNWSF TINARV RAYNAY YSALLQ YHRIIR TDHKNR WGESRE PITSTW DILPVR GSKTRH

PWDLKR THDYTS TITSMD CWRGVR KPHLRP AGDINS MLTFYW SVSSRQ SHRLVW GQRVTT LRDWRP SVMNGR VNEQKC LNQLSL

DVTAWQ VSYGPN FRLGNS HGRPYL HKSFCG GGVRLR SAYLYF YKGRAG NLWGKI LLYSKS NGVVSN LTRNLP GGQTIV FQRLRT

WNLAAF ASSLLE KFMSYA PRYLYI RNLNTN ICGVAT LSSIRR WEVKRE FPKGDR SVLSAL PNVLQT LDSSSK FSYGSW DPRWFD

DYSLVK VIFVNE RDFHGN MEVRYS LNISSN SLVMLE SAALSN WIKYGT RDSGFL QSLYAS TFRLRL IQGNFF FMNELF RPPRLV

AYVTSA INPVLV TESRYV FGMIVF AVQPFR GKVVNL RTVPWV NRAMAK ERVLST VVRWHW FPTSQR NMLVLD STRSLG VLSNHS

RHFWGR FRFMPV MPGKIW IGPQVV DGIFAR MEAVPN CVNHTS IKVQRL FNGSTR SPQHPR WGKGYP FPYKCD QLSWAV CLVSCF

RSDYTL LPFFLK FHRGCP YFNEHT SVQRQR DTLMVT HKFEKL NASQFS RSRWSS TVRLDL TVKRYR RSSMWH PAMFMH SRWTFD

FGLPNG GIMSIG PGKVRN NYYISR DGPRHH KQVLKR ILSNKG ARTVGD VNTVRN GMSRRD NFRTFY RLNPPT VKLVTH QYTSSY

WIHAQA GHMDKR ISTSVE RFKDPL KHTQVP VLSDSA NFAGAV TRIPSS LIVRII TMVGTL RVKLST ADRTMC YEAGYT LLNGRQ

GFYEDV RVVLQL RVQNSW KRPFLP TRMKLR THQRRN GVYNYG SLSYYI TWYAGP STKFGG ILAIDD INCRVC HHSIPS SVFVGL

RVFDGV VHLMSV EANCQK FRFLAS KPAYTS MGAQWT VRYALP ARRNNP NRTLGL TQPYRQ LPLGGF VNRIIR HASRKS IIMNSL

WSNMFS RVDTNR QTLSVY GYQKTQ RICLGT PYGRKS FAGGST GMSLLT VRGDAV RNFKHL CMIDYG NNFWLM AQRLSL FSEYKL

FFHSVQ AGIFPC WTRVLL RYMRQL YDALWL HMVSRD QTNMYM DSMAKS SKLHFT LKQLPL KVSPWL NHLSGL DEWRLG VNVWRL

HYARAV RSAISS LSTFLD WKNRDF KVNLHE LFDRLH WFKNFD LDEVWA RIGVHW AEVAWS KLARTD GTLDLL SVESIR RMGLPW

RLVGEE REREIF MHPTVF NLGSLS TPAESH NHTNVR GSDQQA SILSVL WTYSWT GLHHSA FLVVTN SFYGVN WSGHNW YHTGHS

MDSKFF LRISVH QKQREF YRWFED YRTTFR MHNVTA FGFTDM ALLRYA RHSPLR FLFVGI RIRYGQ TDIRAR DRVQAR STRVAP

TLYFPK AIYTRS HHLFGK GLQLRL LLRNKS GERSGD SVIRFS KMMTMV QRLFLR MDLRMY ATARLL RINVLE HWKLVP RDHRIN

LGKTGG TAGFRV GKGSLT WYDKQS TGGLFR TLKKLS VRYLEN LNMRMI CNFISC GLLDPL IYYENI MKHWSG RGFFYK ITPGRV

SKQRVM SVYLYR FRWNIY SAIFAS GIYYLI EGGFFW LGRYPG NMHVSL SDSGVR TRLMID GKNVYW HMLLTC MYSTFA K*QLIM

NCVSCL VLVRLW FDPILS MHRQTT HRWIKI VWATRG GMFTLP DAFNYR SQVEQV NPKRVL FNHRII AASYFR WSYSSL LRYDYR

YAAIVN VEKARM IFPYHL RYLIPD SRVGYD SWVFDL RWSYFG YPPISG DRKGTM KSTIPS QKWGIQ NLNAAK FHWASL QWDSRF

NMSATP WTKSTS KYVSIF QWWNSN MKPLGC GLTIIP HDMISI HLCIVE SGSNVI VIGRWY MKVGVH RARSPD NRSFIS TSFILM

RPLHLH RNMHWN CQETKW IDLTWH NTLRRT FWIATR SVYSYY WGIQIP LGIMWT HREENA NTQLVH CIENDY GIHQWI LTIPAL

KRSVEL YSNHTE KDGMPQ VTFIKP IHVSVH RNGRWL YSMKLM GCVHSS SKGYHG LENKMN SLIKGV AIWQAL YDPGAI IIDRTT

MWRPYF SQMGSH SHKVPL KNSMFR IKIKPR MLWQLS FALARA ATNQWA KHWAEN ISTETY TGMWLC KRSPVL ASPTLL RTRDKH

GDYRVY SFNKVN ALKWVG FWHAAL PDVKFS RLYATN LDLTMR LWSSGR SSAEGR TKYMLS NSAGYT NGAVGM LADHQK RWVQKY

FLHWVG LPWEHS HLKTQV TRTIYV QGLRTR MQSTMI TSFANY FLAKLR DKTSVR WKMNNL RTLTNL SMRMPR SLSSKI ISSVIA

SNTWIL YSPVYA TPQSTT QMMKRS PSRHSP WPSEH* GHSAWS YDRYFD GKHGVI ALQGVG RSCQVF SAFRTT TLVRGQ VQSGNL

LMISCA ARVMRT RVRANN RFPKQR SINIRA ISSVLW SLGFNV DAGAFY KGGSSL FFNSSC KAQTYG IPRHDG GASKTP QMSASV

NMKFIM YLNTVI TSSLQL RVTAKS LPPGAH RWTGNM IIMLGR SVFNNV QNPLQY FAEARG MYFFLA RDSWWWAMHISR LSPNDR

RVRTTM CTLRAV QILRAA RSLKIP DLTVMN HDKRQI MLSTRL GAKFNM MTADHN TTIRLQ RSGYLI LLRHLI KITGLE YRYITT

RDSNIV EQESCA LMTKLA ISPFEQ WPLTKK YDMCIV TISPNA RRLEFM HKQLTH TTHELF LWYQRN YHGRPR GQYHRQ SLWKSH

AYPYVH VKTFQT YFSYEP YIYEMK SLRNSR GWCSKS RHYMQQ WKCKNH QVTNSG NFMSRV KSLEVI VRCCKK TMGYCV NLKYRR

RSGKLH KNGTNQ QHWNVK VNLAVI AYSYIL TAKNPM GPGNLL VSTPVY LSSGIL PMQVNY SPQLGH KLLRLH TFSIWV MMMEVD

GHMDRI RPMWYL QASVFR RAIGLK QFYAYD NFKKYH QLVTMM LDSKRP SYMQYR LNPLIT VAAMFK MGHQAI LKCCTY QASSFT

AHRRSL IQLVRI YRQWRN ISTCKW RKGNKS RSAKID FLPMRG IYTGVL KTVPSG SMNVDS DINTEQ KAALST RRSSST MIRSSV

KLENPA PQGPPL GFRPGN VHFEWY GPRVSR STVVLS VHVEHV RSFIKQ SDSKTQ HGSDCL VQCQSS SSSFFA MVTYHL SGFDVR

RDRLGF TGVAHI ARLPVL YSNRGY MYRWFD WDINLH TYYGVT KVNRVD FNFEVR IGKSLD
