# Supplementary material for: CleavPredict: A Platform for Reasoning about Matrix Metalloproteinases Proteolytic Events
Source: PLoS One. 2015 May 21;10(5):e0127877. doi: 10.1371/journal.pone.0127877 (PMC4440711; doi:10.1371/journal.pone.0127877)
Supplement: S3 Table — The header of each matrix contains the values of offset and threshold. The offset values are already incorporated into the log2 PWM matrices in appropriate positions where given amino acid is not observed in phage display substrates. (DOC) [file pone.0127877.s005.doc]

**S3 Table.** Final log_2_ PWM matrices for 11 MMPs. Appropriate offset and thresholds are given in the matrices headers.

MMP17 -5.5 0.6 (offset, thresh) MMP25 -6.0 -0.3 (offset, thresh)

P3 P2 P1 P1' P2' P3 P2 P1 P1' P2'

G -3.94053 0.57570 -1.33588 -5.50000 -2.16036 G -2.17097 0.99158 0.17060 -6.00000 -0.70094

A 1.33248 1.01329 1.20491 -5.50000 -1.30910 A 2.05469 1.22642 -0.07620 -2.87133 -2.01963

P 2.45553 -5.50000 -0.13003 -5.50000 -5.50000 P 2.40402 -6.00000 1.05452 -6.00000 -6.00000

C -5.50000 -5.50000 -1.76263 -5.50000 -1.14407 C -6.00000 -6.00000 -0.99312 -6.00000 -6.00000

T -1.38022 -2.92292 -0.70282 -5.50000 0.92708 T -1.61069 -3.15341 -2.10320 -6.00000 0.29905

S -1.25246 0.36378 0.50483 -2.85767 -0.26961 S 0.28258 0.03376 -0.18508 -6.00000 1.14542

D -5.50000 -1.10603 -1.29590 -5.50000 -2.23922 D -6.00000 -0.33651 0.88863 -6.00000 -6.00000

N -2.66749 -1.09117 1.17084 -5.50000 -0.10612 N -2.89797 0.67834 -0.64460 -6.00000 -1.03901

E -5.50000 0.28071 0.03005 -5.50000 -5.50000 E -1.67056 -0.17214 -1.78537 -6.00000 0.28297

Q -2.10494 1.00378 0.54040 -0.02575 -0.17008 Q -1.33540 0.49318 0.77940 -2.42616 0.38245

K -5.50000 -1.28531 0.09564 -5.50000 0.87793 K -6.00000 -0.51579 -0.25032 -6.00000 0.16743

H -2.13783 0.99254 -1.37474 -5.50000 -0.71437 H -0.78334 -0.00348 1.13171 -6.00000 -0.42486

R -5.50000 -2.48883 0.27810 -5.50000 0.74045 R -6.00000 -1.71928 0.88412 -6.00000 0.02995

V 1.41311 -0.92497 -5.50000 -3.17814 0.45407 V 1.41395 -3.32539 -3.38383 -2.40866 1.00661

I 1.05119 -0.95814 -5.50000 -1.28005 0.67149 I 0.23575 -6.00000 -6.00000 1.25498 -0.45403

L 1.32812 0.46463 -0.04899 2.85592 0.34015 L -0.09875 0.84713 -0.40501 2.84242 -1.24482

M 0.51534 1.48747 0.85338 2.25951 0.73041 M 0.64743 0.36391 0.20786 1.27900 1.11944

F -1.62941 0.28904 -0.41513 -2.54852 -1.87445 F -1.27494 -0.74879 0.35438 -1.19409 0.00000

Y -2.07503 0.08538 -0.04899 -1.19915 0.16562 Y -2.89046 -0.84554 0.30549 -1.75156 0.84744

W -3.24236 0.38235 0.09661 -5.50000 -1.59152 W -6.00000 1.65436 -0.13387 -2.51053 -1.71705

MMP2 -5.0 0.3 (offset, thresh) MMP9 -4.0 1.5 (offset, thresh)

P3 P2 P1 P1' P2' P3 P2 P1 P1' P2'

G -3.62433 1.06884 0.39541 -5.00000 -5.00000 G -3.85266 -3.85998 1.07386 -4.00000 -2.53742

A 0.01648 2.17755 1.98511 -5.00000 -0.43465 A 1.10996 -0.08330 1.75667 -4.00000 -1.27114

P 3.67323 -5.00000 0.18623 -5.00000 -5.00000 P 3.88152 -4.00000 0.10978 -4.00000 -4.00000

C -5.00000 -5.00000 -5.00000 -5.00000 -5.00000 C -4.00000 -4.00000 -4.00000 -4.00000 -4.00000

T -3.64891 -1.28468 -0.55649 -5.00000 -0.11598 T -2.29236 -1.83505 -2.78498 -4.00000 1.16299

S -5.00000 1.29671 0.47713 -5.00000 0.31534 S -2.90163 -0.28535 0.30314 -2.02691 -1.10612

D -5.00000 -5.00000 -1.56460 -5.00000 -0.77981 D -4.00000 -4.00000 -0.79305 -4.00000 -4.00000

N -5.00000 -1.77492 0.82814 -5.00000 -5.00000 N -3.57966 -1.58831 1.35177 -2.64566 -4.00000

E -5.00000 -5.00000 0.34631 -5.00000 -5.00000 E -4.00000 -1.43881 -1.46709 -4.00000 -0.96857

Q -5.00000 0.23257 -0.03642 -5.00000 -5.00000 Q -4.00000 -1.41088 -0.75030 -1.51684 0.13091

K -5.00000 -2.29095 -0.11861 -5.00000 2.07433 K -4.00000 0.94001 -0.51696 -4.00000 -0.08410

H -1.23657 -1.77868 0.52645 -5.00000 -5.00000 H -3.05002 -2.00711 0.71305 -0.89671 0.90855

R -4.18416 0.73434 -0.62802 -5.00000 1.78484 R -4.41273 1.99132 -1.85647 -3.48164 1.42227

V 0.45373 -0.45670 -5.00000 -0.53290 -0.99337 V 1.46922 -0.54765 -3.06552 -0.08431 -0.24492

I 0.95241 -1.22684 -5.00000 0.04328 1.35331 I -1.86097 -1.87033 -4.00000 0.49187 0.51681

L 1.18495 -0.13664 -1.14777 2.49665 -1.07488 L -0.55807 0.32296 -0.84570 2.36104 -1.49634

M -0.16839 -1.89668 -2.83033 1.89696 0.38247 M -2.20416 -0.31778 -1.47381 1.72888 1.13094

F -0.99125 -2.20204 -0.26879 1.02271 -5.00000 F -3.54153 -0.10856 0.50276 -0.13276 -0.66657

Y -5.00000 -5.00000 0.38274 0.80225 -1.37493 Y -4.00000 0.28011 -1.22421 1.27321 0.95847

W -5.00000 -2.88629 0.41286 -0.37175 -1.13210 W -0.83266 0.69256 1.32193 -1.60121 0.35332

MMP14 -5.5 0.5 (offset, thresh) MMP15 -2.5 1.3 (offset, thresh)

P3 P2 P1 P1' P2' P3 P2 P1 P1' P2'

G -5.50000 0.14083 1.75280 -5.50000 -2.31983 G -0.49872 -0.33613 0.84287 -2.50000 -2.50000

A 0.74707 1.23573 1.75753 -5.50000 -2.05355 A 0.99000 1.27066 1.11064 -3.19905 -0.12979

P 3.17886 -3.07850 0.30329 -5.50000 -5.50000 P 2.02738 -2.50000 0.14183 -2.50000 -2.50000

C -1.19350 -5.50000 -5.50000 -5.50000 -5.50000 C -2.50000 -2.50000 -2.50000 -2.50000 -2.50000

T -3.68390 -1.64155 -3.59144 -5.50000 -0.31985 T -0.20146 -2.50000 -2.50000 -2.50000 -1.98102

S -0.97114 -0.80233 0.69595 -5.50000 0.28140 S 0.11531 0.29100 0.29454 -4.00092 -2.54970

D -5.50000 -5.50000 -2.59955 -5.50000 -5.50000 D -2.50000 -1.07926 -1.43908 -2.50000 -2.50000

N -5.50000 -0.39480 1.06884 -5.50000 -5.50000 N -3.22566 -0.06442 1.40618 -2.50000 -2.50000

E -5.50000 0.56205 -5.50000 -5.50000 -5.50000 E -1.99827 -2.08485 -2.11309 -2.50000 -0.41217

Q -5.50000 -1.80233 -2.87874 -2.91430 0.34850 Q -0.07816 0.35810 -1.13327 -1.75389 1.49466

K -5.50000 -2.32590 -3.32351 -5.50000 -0.28154 K -2.50000 -1.58048 -3.16294 -2.50000 1.47229

H -1.27153 0.99376 2.22848 -5.50000 -1.45882 H -2.69600 -2.65317 2.18250 -2.50000 -1.12001

R -4.21920 1.43637 -5.50000 -5.50000 1.69644 R -4.05865 0.70786 -4.08754 -2.50000 0.65673

V 1.21526 -1.22860 -5.50000 -5.50000 -2.61228 V 1.18932 -2.65308 -2.50000 -2.15142 0.31146

I -0.25244 -1.26177 -5.50000 1.13876 0.73440 I -0.28461 -1.51634 -2.50000 1.08772 1.07320

L 0.15001 -0.01958 -5.50000 2.86359 0.72124 L 0.23648 0.94824 -2.60717 2.76467 -0.35499

M 0.44875 0.97525 0.71968 0.65330 1.34853 M 0.73474 0.39875 0.61711 0.81377 0.68733

F -0.54075 -3.23704 -0.13379 0.05469 -0.44898 F -0.60267 0.24541 0.17867 -0.78485 -1.69516

Y 0.32177 0.47362 -0.35262 -1.23977 -1.99383 Y -0.21819 0.91419 1.05576 0.24263 1.92990

W 0.62386 0.66368 -0.62206 -0.99873 -0.16604 W 0.78433 0.04653 1.21647 -0.83828 -1.41223

MMP16 -5.0 0.3 (offset, thresh) MMP24 -5.5 0.6 (offset, thresh)

P3 P2 P1 P1' P2' P3 P2 P1 P1' P2'

G -1.94619 -0.49408 0.71734 -5.00000 -5.00000 G -1.86470 -1.87207 0.32488 -5.50000 -2.30300

A 1.27950 1.36377 0.92622 -5.00000 -2.18186 A 2.06879 1.53270 1.59264 -5.50000 -0.45172

P 2.67614 -5.00000 0.15380 -5.00000 -5.00000 P 2.80341 -5.50000 0.09777 -5.50000 -5.50000

C -5.00000 -5.00000 -5.00000 -5.00000 -5.00000 C -5.50000 -1.53144 -5.50000 -5.50000 -5.50000

T -5.00000 -1.60660 -5.00000 -5.00000 0.87375 T -0.88939 -5.50000 -5.50000 -5.50000 -2.30300

S -1.41016 -0.02520 0.84709 -5.00000 -0.01684 S -1.69124 -1.29739 1.05979 -5.50000 -0.28669

D -5.00000 -5.00000 -5.00000 -5.00000 -5.00000 D -5.50000 -5.50000 -5.50000 -5.50000 -5.50000

N -5.00000 -0.68179 1.58021 -5.00000 -5.00000 N -2.59170 -0.79298 1.19772 -5.50000 -5.50000

E -5.00000 0.05266 -5.00000 -5.00000 -0.87930 E -5.50000 -2.45087 -5.50000 -5.50000 -5.50000

Q -5.00000 0.37007 -0.58074 -5.00000 1.02754 Q -5.50000 0.99211 -3.08424 -5.50000 0.68731

K -5.00000 -1.29095 -5.00000 -5.00000 1.32709 K -3.62148 -5.50000 -5.50000 -5.50000 1.15036

H -0.55851 -2.10060 2.49403 -5.00000 -1.58714 H -0.06200 0.30280 2.50838 -5.50000 0.14302

R -2.50610 1.14937 -5.00000 -5.00000 1.67503 R -5.50000 1.23084 -4.45355 -5.50000 2.22233

V 1.16928 -2.10054 -5.00000 -1.85626 -1.15566 V 1.25074 -1.01911 -5.50000 -4.10234 -5.50000

I -1.53943 -1.54877 -3.50881 0.30489 -0.20127 I 0.34938 -1.88235 -5.50000 1.11766 -5.50000

L 0.71101 0.32569 -3.63953 2.65353 -2.40705 L -0.12951 1.18088 -3.55809 2.83810 0.19755

M -0.97573 -0.41127 1.01765 2.57361 1.80517 M -1.21621 0.86283 0.25111 1.82629 1.36541

F 0.75722 0.79795 -0.42080 -1.96358 -2.16229 F -0.96866 -0.12057 0.66067 -1.88779 -0.43210

Y 0.58226 0.28616 0.53030 -1.52110 0.87782 Y 0.11624 -0.73190 -0.75081 -1.44528 -1.97698

W 0.75191 1.18403 0.67590 0.62683 -1.87933 W -1.58164 0.45815 1.24279 -1.61931 -5.50000

MMP3 -5.0 1.5 (offset, thresh) MMP10 -5.0 1.5 (offset, thresh)

P3 P2 P1 P1' P2' P3 P2 P1 P1' P2'

G -5.00000 -1.32452 -1.66008 -5.00000 -5.00000 G -2.67291 -3.68019 -0.65326 -5.00000 -5.00000

A 0.00161 1.70549 2.20972 -4.18731 -0.66072 A 2.03819 -0.77801 1.82396 -5.00000 -5.00000

P 3.98838 -3.90638 -0.26158 -5.00000 -5.00000 P 4.13475 -3.09221 0.13755 -5.00000 -5.00000

C -5.00000 -5.00000 -5.00000 -5.00000 -5.00000 C -5.00000 -5.00000 -1.49504 -5.00000 -5.00000

T -5.00000 -5.00000 -0.41933 -5.00000 0.24291 T -5.00000 -5.00000 -1.28324 -5.00000 -0.93798

S -5.00000 -1.38926 1.06359 -4.98942 -0.32575 S -3.30685 -1.95360 1.18745 -3.17514 0.07830

D -5.00000 -5.00000 -2.42747 -5.00000 -5.00000 D -5.00000 -5.00000 -1.61329 -5.00000 -5.00000

N -5.00000 -5.00000 -0.28263 -5.00000 -1.68010 N -5.00000 -5.00000 0.23197 -5.00000 -5.00000

E -5.00000 -3.07327 1.29082 -5.00000 -5.00000 E -5.00000 -5.00000 -0.28732 -5.00000 -5.00000

Q -5.00000 0.61765 1.15135 -1.74226 -2.25863 Q -5.00000 -0.81608 0.56698 -2.92807 -0.26961

K -5.00000 -5.00000 -5.00000 -5.00000 0.84826 K -3.42973 -5.00000 -1.33719 -5.00000 0.51535

H -5.00000 0.16584 -0.33639 -2.53717 -2.06594 H -5.00000 -2.82735 0.11522 -5.00000 0.92305

R -5.04692 -5.00000 -2.75389 -5.00000 1.55877 R -4.23270 -4.22135 -2.26167 -5.00000 -0.03716

V 1.47224 -2.31955 -4.69974 -1.40285 0.58790 V 0.06402 -5.00000 -5.00000 -3.91050 -1.23043

I 1.20504 -1.50471 -5.00000 1.52384 1.31989 I -3.26617 -1.27550 -5.00000 0.98752 -0.69108

L -0.75190 0.73747 -0.59557 2.55207 -0.07856 L -4.18573 1.83458 0.02595 2.05387 0.10311

M -5.00000 0.24044 0.69911 -2.49654 0.47834 M -1.43944 1.57818 1.29090 2.56557 2.73040

F -5.00000 2.36136 1.11628 1.33111 -1.05613 F -3.36186 2.44971 0.26750 1.47391 0.34792

Y -5.00000 0.79254 -0.37319 0.88651 -0.60101 Y -3.39244 0.55941 -0.19644 1.60447 1.70994

W -5.00000 1.92322 -5.00000 -5.00000 -0.77322 W -5.00000 1.70883 -1.95774 -2.01246 0.63085

MMP8 -3.0 1.2 (offset, thresh)

P3 P2 P1 P1' P2'

G -0.60719 -0.93644 -0.58751 -3.00000 -1.59400

A 1.03354 -0.29721 0.44579 -2.04441 -3.00000

P 2.80511 -3.00000 0.17088 -3.00000 -3.00000

C -3.00000 -3.00000 -3.00000 -3.00000 -3.00000

T -0.04689 -2.32648 -0.40192 -3.00000 0.99093

S 0.13743 -0.20976 0.37873 -2.10935 -1.16266

D -2.03694 -2.09461 0.45244 -3.00000 -3.00000

N -0.48618 -0.49480 0.73479 -3.00000 -0.34713

E -0.84370 -1.51523 0.62642 -3.00000 -3.00000

Q -1.09351 1.62816 -0.56366 -0.59930 0.07431

K -1.68584 -2.01089 -0.13395 -3.00000 0.44425

H -0.54145 0.23840 1.22160 -2.97914 -3.00000

R -2.16708 -0.89244 0.12600 -3.00000 0.89171

V 1.11041 -1.49854 -3.14190 -1.58182 1.11357

I -0.20043 -1.53169 -3.00000 1.16430 -0.34713

L -0.74139 0.59803 0.02130 2.24409 0.03200

M -0.95867 1.04637 -0.55023 1.52089 2.07432

F -1.29608 1.24798 0.81867 0.63277 -1.30819

Y -2.06365 1.52562 0.28433 1.69976 0.31694

W -1.64606 1.05672 -1.62896 -1.68371 -1.02517
