# Supplementary material for: CleavPredict: A Platform for Reasoning about Matrix Metalloproteinases Proteolytic Events
Source: PLoS One. 2015 May 21;10(5):e0127877. doi: 10.1371/journal.pone.0127877 (PMC4440711; doi:10.1371/journal.pone.0127877)
Supplement: S4 Table — The calculations have been performed to establish the optimal values for threshold and offset parameters that are implemented in the CleavPredict web server for predicting cleavage sites in proteins. For each average value the sample standard deviations is provided. For abbreviations see Table 1. (DOC) [file pone.0127877.s006.doc]

**S4 Table**. Average values for sensitivity, specificity, accuracy, precision, Matthews correlation coefficients, false positive rate, true positive rate and optimal values for threshold and offset from the 10-fold cross-validation using approximately two-third of the entire sets (internal test) of available substrates for every MMP. The calculations have been performed to establish the optimal values for threshold and offset parameters that are implemented in the CleavPredict web server for predicting cleavage sites in proteins. For each average value the sample standard deviations is provided. For abbreviations see Table 1.

|  | **Sensitivity**  **(TPR)**  **(%)** | **Specificity**  **(%)** | **Accuracy**  **(%)** | **Precision**  **(%)** | **MCC** | **FPR**  **(%)** | **F1** | **threshold/ offset** |
| --- | --- | --- | --- | --- | --- | --- | --- | --- |
| MMP2 | 86.8±6.4 | 95.7±2.7 | 93.9±2.8 | 84.5±7.8 | 0.82±0.07 | 4.3±2.7 | 0.85±0.05 | 0.9 / -6.0 |
| MMP9 | 86.9±7.3 | 96.7±1.9 | 94.9±2.7 | 85.2±8.6 | 0.83±0.09 | 2.6±1.1 | 0.86±0.07 | 1.5 / -3.5 |
| MMP14 | 86.4±5.0 | 94.8±3.3 | 93.1±2.7 | 82.6±9.0 | 0.80±0.07 | 5.2±3.3 | 0.84±0.04 | 0.8 / -6.5 |
| MMP15 | 83.7±8.0 | 92.6±3.8 | 90.4±2.7 | 78.2±9.6 | 0.75±0.06 | 7.4±3.8 | 0.80±0.05 | 0.4 / -3.5 |
| MMP16 | 86.1±6.4 | 97.3±1.3 | 95.0±1.6 | 88.7±5.2 | 0.84±0.05 | 2.7±1.3 | 0.87±0.04 | 1.2 / -4.5 |
| MMP24 | 88.7±7.2 | 95.1±2.1 | 93.9±1.9 | 82.3±5.5 | 0.82±0.06 | 4.9±2.1 | 0.85±0.04 | 1.7 / -4.5 |
| MMP17 | 90.2±6.6 | 90.4±4.8 | 90.4±2.9 | 75.9±8.3 | 0.77±0.06 | 9.6±4.8 | 0.82±0.04 | 0.7 / -5.0 |
| MMP25 | 86.3±5.5 | 88.7±4.2 | 88.0±2.7 | 69.5±5.5 | 0.70±0.06 | 11.3±4.2 | 0.77±0.05 | -0.4/ -6.0 |
| MMP3 | 96.8±3.4 | 93.6±1.3 | 94.1±1.2 | 75.3±4.0 | 0.82±0.04 | 6.4±1.3 | 0.85±0.03 | 1.5 / -4.0 |
| MMP8 | 91.0±5.7 | 84.7±3.0 | 85.7±2.8 | 54.6±5.7 | 0.63±0.06 | 15.3±3.0 | 0.68±0.05 | 0.3 / -3.0 |
| MMP10 | 94.1±8.5 | 89.8±3.4 | 90.5±3.1 | 65.5±7.3 | 0.73±0.08 | 10.2±3.4 | 0.77±0.06 | 1.5 / -5.0 |
